# Single-cell transcriptomic analysis identified resistant MDSCs and a stress-tolerant gene co-expression network as common MDSC features across multiple disease settings

**DOI:** 10.3389/fimmu.2025.1565211

**Published:** 2025-04-08

**Authors:** Tianmeng Chen, Julia Hughes, Alyssa Gregory, Julia Conroy, Patricia Loughran, Jinming Song, Wei Chen, Timothy Billiar

**Affiliations:** ^1^ Department of Surgery, University of Pittsburgh, Pittsburgh, PA, United States; ^2^ Department of Pathology, H. Lee Moffitt Cancer Center and Research Institute, Tampa, FL, United States; ^3^ Department of Pediatrics, University of Pittsburgh, Pittsburgh, PA, United States

**Keywords:** myeloid derived suppressor cells, MDSCs, cellular stress, scRNA-seq, stress tolerant

## Abstract

**Background:**

Myeloid-derived suppressor cells (MDSCs) are a heterogeneous population of immunosuppressive myeloid cells. The identification of a molecular signature common to MDSC regardless of tissue source would aid in the classification of cells as MDSCs.

**Methods:**

Single-cell RNA sequencing (scRNA-seq) was performed on GM-CSF+ IL-6-induced human MDSCs to characterize the extent of heterogeneity within monocytic MDSCs (M-MDSCs). Cytokine-treated PBMCs were also cultured in the absence of serum to include an additional element of cell stress. Independent published bulk and single-cell transcriptomic datasets were used for validation.

**Findings:**

A cluster of cells with preserved MDSC features was induced by the combination of inflammatory signals and cell stress in the form of serum starvation (resistant MDSCs, rMDSCs). A gene co-expression module (the yellow module) was identified specific to rMDSCs. The genes upregulated in MDSCs can be further classified into stress-tolerant vs. -sensitive features. This yellow module mostly contained stress-tolerant genes and showed excellent separation for distinguishing M-MDSCs from control cells across a range of *in vitro* and *in vivo* conditions (ROC AUC = 0.954), a feature not found in the stress-sensitive genes. Importantly, rMDSCs were identified in scRNA-seq datasets of immune cells from multiple human cancer types. Tumor C1Q macrophages, which have been associated with immunosuppression, highly expressed the yellow module gene signature.

**Interpretation:**

These results demonstrate the importance of the combined roles of inflammation and cellular stress in shaping the features of M-MDSCs and highlight cellular resilience represented by rMDSCs and the role of stress-tolerant features in defining common MDSC features.

## Introduction

Myeloid-derived suppressor cells (MDSCs) are a heterogeneous population of myeloid cells defined by their immunosuppressive effects on T cells. The prevalence of MDSCs dramatically rises during acute (i.e., trauma and sepsis) and chronic (i.e., cancer) inflammatory diseases (1–3) and is thought to contribute to infectious complications and poor prognosis by suppressing immune responses (4–7). MDSCs can be generally classified by lineage as either granulocytic (G-) or monocytic (M-). Human MDSCs are defined as CD11b+CD33+HLA-DR^lo^ (G-MDSCs: CD66b+/CD15+ vs. M-MDSCs: CD14+) (8).

Transcriptomic sequencing has been used to study the features of MDSC isolated from patients with different diseases. An scRNA-seq study sequenced tumor and adjacent normal tissues from patients with non-small cell lung cancer and demonstrated that MDSCs are distinct from M1 and M2 macrophages in their transcriptomic profiles (9). Another scRNA-seq study identified CD84 as a novel surface marker to enrich for MDSCs in human breast cancer (10). A study combining experimental and RNA-seq data demonstrated impaired phagocytosis in M-MDSCs isolated from patients with acute-on-chronic liver failure due to Toll-like receptor pathway suppression (11). However, the identification of features, including molecular markers common across human MDSC populations, remains limited. Two major limitations need to be addressed (1): First, the markers and transcriptomic signatures are often dependent on the source of the MDSCs. The characterization of MDSC features from one source is not always generalizable across multiple sources of MDSCs. Second, the MDSC markers/signatures typically overlap with those from conventional immune cell populations leading to challenges in establishing thresholds to distinguish MDSCs.

As a strategy to identify heterogeneity within human MDSCs, we applied single-cell analysis combined with flow cytometry and functional assays on monocytic MDSCs generated by the exposure of PBMC to GM-CSF + IL-6 *in vitro*. Because MDSCs likely face further selection pressure by the cellular stress encountered within harsh microenvironments such as those found in tumors or inflammatory conditions, we also exposed the cytokine-generated MDSCs to serum starvation. This led us to identify and characterize a cluster of resistant MDSCs (rMDSCs) that retained MDSC function and had gene expression and cell surface markers unique from the other clusters under serum starvation. Gene coexpression network analysis revealed a gene module in rMDSCs, which was stress-tolerant. The gene module identified MDSC with high specificity across a number of MDSC sources including within multiple solid malignancies and, interestingly, was also identifiable in AML. These findings suggest that the resistance to cell stress is likely to be a factor that shapes the transcriptome and function of monocytic MDSCs.

## Methods

### Human M-MDSC generation *in vitro*

Cryopreserved human PBMCs obtained from healthy volunteers enrolled in a protocol approved by the University of Pittsburgh Institutional Review Board (#19040329) were used for these studies. All participants gave written informed consent. PBMCs were also purchased from STEMCELL (**Supplementary Table S1**). MDSCs were generated by treating PBMCs with GM-CSF (10 ng/mL) + IL-6 (10 ng/mL) (10, 12) at 5 × 10^5^ cell/mL in complete media at 37° with 5% CO_2_. Cytokines were added daily for 3 days. PBMCs cultured in complete media alone were used as control. Serum starvation was performed using serum-free media.

### T-cell suppression assay

Human T cells were stained with CellTrace Far Red dye with 1 µL of 1 µM dye per 5 × 10^5^ cells, at RT for 20 min. T cells were seeded 5 × 10^4^ cells/well in a 96-well plate. Flow sorted control monocytes (CD33+, C group), MDSCs (CD33+, T group), or subsets (CD52^hi^CD14^lo^ vs. CD52^lo^CD14^hi^ in CD33^lo^CD11b+ cells after serum starvation) were added to T cells in a 1:1 or 1:2 M:T ratio (labeled in each figure legend). Human CD3/CD28 dynabeads were added at a bead-to-cell ratio of 1:1 to stimulate T-cell proliferation. T cells cultured with CD3/CD28 alone acted as a positive control. For each donor, duplicates were used for each experimental condition. Cells were cocultured for 4 days, and then harvested, stained for anti-human CD3 SuperBright 600 and live/dead dye, and evaluated by flow cytometry. Cells were cultured for 4 days, and then harvested, stained for anti-human CD3 SuperBright 600 and live/dead dye, and evaluated by flow cytometry. For each MDSC population, the assay was evaluated in at least three different donors.

### Phagocytosis assay using flow sorted cells

pHrodo™ Green E. coli BioParticles™ Conjugate was purchased to evaluate the capabilities of phagocytosis. One vial of BioParticles (2 mg) was resuspend in 2-mL full media to make a 2-mg/mL BioParticle solution. Then, BioParticle solution was added to flow-sorted cells (CD52^hi^CD14^lo^ and CD52^lo^CD14^hi^) at 1:10 for a final concentration of 0.1 mg/mL in full media. Cells and BioParticles were incubated for 30 min at 37°C, 5% C0_2_ and then placed on ice to stop the reaction, with technical duplicates for each population. Cells incubated under the same experimental condition without BioParticles added were used as negative control. After one wash, the cells were analyzed using flow cytometer in FITC channel.

### Single-cell library preparation, sequencing, and analysis

We followed Chromium Next GEM Single Cell Multiome ATAC + Gene Expression protocol (CG000338 Rev C) and Chromium Single Cell 3′ Reagent Kits User Guide (v3.1, CG000315) to prepare the corresponding libraries. Libraries were pair-end sequenced in UPMC Genomic Center. The 10x Genomics Cell Ranger pipeline (13, 14), Seurat (v4.0.5) (15), and Signac (v1.4.0) (16) were used to analyze single-cell data. Details are described in supplemental methods.

### Gene set enrichment analysis

A group of significant genes were ranked by a statistical estimate (e.g., correlation coefficient) and used as the input. Gene set enrichment analysis (GSEA) was performed using the fgsea R package (v1.10.1) via fgseaMultilevel() function. MSigDB gene sets v7.4 were used.

### Identification of hdWGCNA modules

We identified consensus co-expression networks using hdWGCNA (17, 18) across the two subjects of the scRNA-seq data we generated, following the tutorial of https://smorabit.github.io/hdWGCNA/articles/consensus_wgcna.html. Generally, the co-expression network was constructed in each individual separately, and then the networks were integrated, and the gene modules were identified.

### Analysis of the bulk MDSC dataset

The bulk MDSC dataset, including five different sources of MDSCs along with the CD11b+ counterparts isolated from healthy spleen from or bone marrow from the same strain of mice, was obtained from the published dataset GSE21927 (19). For each signature, the signature score in each sample was calculated by the average expression of the signature genes after z-score transformation across all the samples. To calculate the AUC (area under the curve) of ROC (receiver operating characteristic) curve for each signature, logistic regression was fitted between the signature scores and the MDSC identity. Then, the ROC curve was built using roc() function from the pROC package (v1.18.5).

### Analysis of the tumor immune atlas scRNA-seq dataset

This data resource integrated published datasets from 13 cancer types involving 217 patients (20). The Seurat object (TICAtlas.rds) and the metadata (TICAtlas_metadata.csv) were downloaded from the website (https://zenodo.org/records/5205544). There are two different annotations (lv1_annot and lv2_annot) in the meta data. We extracted the cells which were annotated as monocytes or macrophages in both annotations and excluded the proliferating cells (annotated as “Macro. and mono. prolif.,” the cells containing mixed populations). In the manuscript, we used lv1_annot to show the cell subset defined by the original paper. After extracting the monocytes and macrophages, the data were re-normalized and scaled using the Seurat package. We used our scRNA-seq dataset as reference. The cell annotations were transferred to this dataset using the Seurat package. The details of label transfer and signature score calculation were mentioned under the session of “Single-cell Feature-barcode count matrix processing”.

### Analysis of the AML scRNA-seq dataset

The UMI count matrix and the meta data of all the samples were downloaded from GEO (GSE116256). The data from different samples were catenated, normalized, and scaled using the Seurat package. Signature scores were calculated as mentioned under the session of “Single-cell Feature-barcode count matrix processing”.

### Analysis of the TCGA AML dataset

#### Data processing

TCGA-AML (RNA-seq) level 3 gene expression data were downloaded from https://gdac.broadinstitute.org/runs/stddata:2015_02_04/data/LAML/20150204/. RPKM data were extracted, log2(RPKM + 1) transformed, and proceeded with downstream analysis. The patient clinical data and mutation annotations were obtained from the original paper (21). For the TCGA-AML data set, we focused on the well-established mutations of 14 genes that have been associated with AML (21, 22), including TP53, NPM1, FLT3, DNMT3A, IDH1, IDH2, NRAS, KRAS, RUNX1, TET2, CEBPA, WT1, PTPN11, and KIT. We annotated the patients with non-silent mutations as mutation positive, and the patients with wild-type genes or silent mutations as negative.

#### Time-to-event analysis in the TCGA-AML dataset

Wilcoxon rank-sum test was performed for each mutation mentioned above, to identify the mutations significantly associated with the expression of the yellow module. The significant mutations (p < 0.05) were included in the Cox regression model along with age, gender, cytogenetic risk category, FAB morphology code, and blast cell percentage. The Cox proportional hazards model was performed by coxph() function in R using the survival package (v3.1.8). We evaluated time-to-relapse and time-to-death separately.

## Results

As an overview of the methods used in this study, the major steps are summarized as follows. We carried out *in vitro* culture experiments exposing human PBMCs to GM-CSF + IL-6 stimulation, with or without serum starvation. The cells were then subjected to scRNA-seq. This led to the identification of a subset of MDSCs resistant to the harsh microenvironmental conditions created by serum starvation. These resistant MDSCs (rMDSCs) were characterized further by gene co-expression network analysis, which yielded a gene co-expression module (the yellow module) unique to the rMDSC. The yellow module signature was used to query published MDSC-relevant datasets representing multiple diseases and tissues to establish the signature as a feature common to diverse populations of MDSC.

### scRNA-seq reveals that DNA damage response is a feature of *in vitro*-induced MDSCs

**Figure 1:**
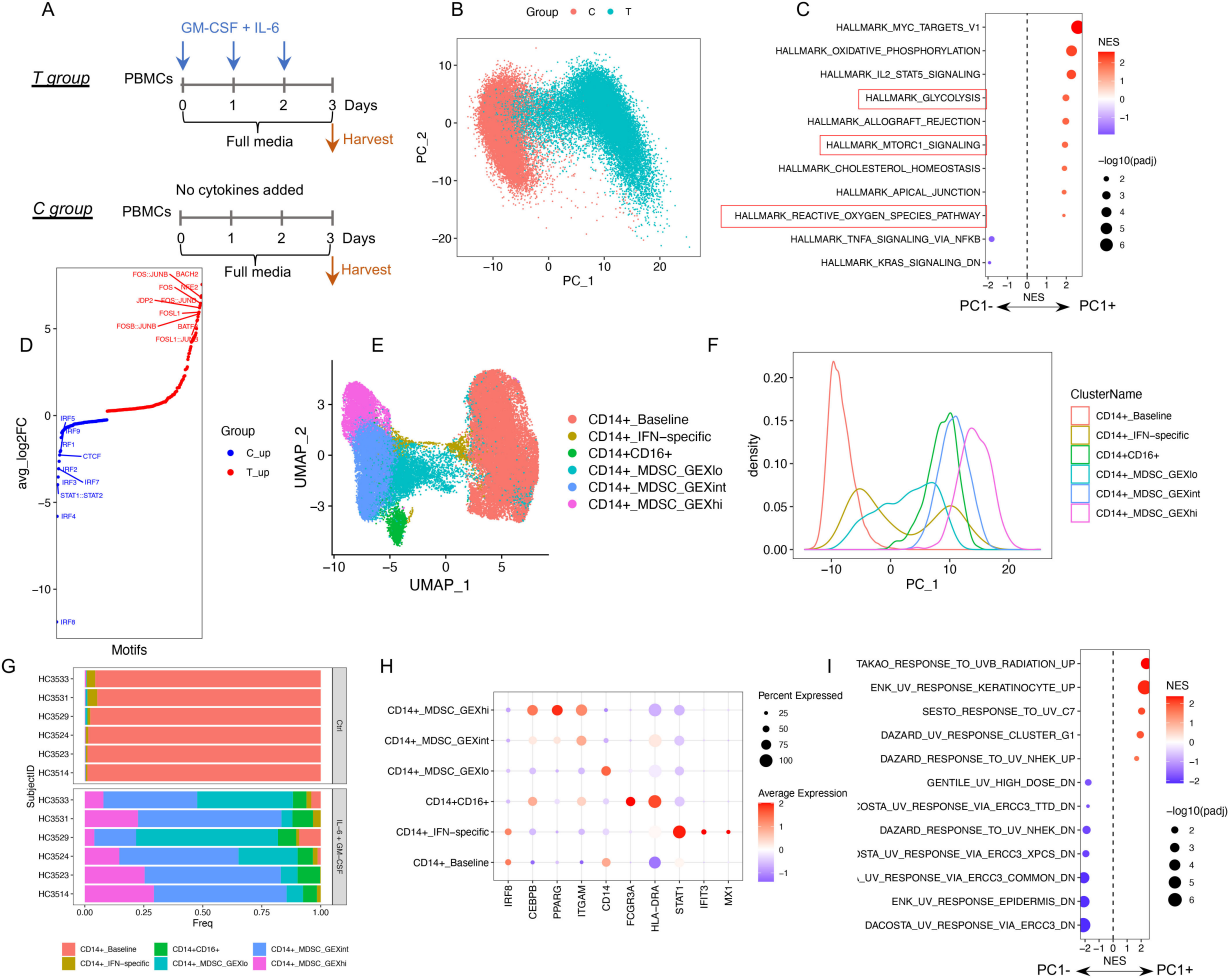
The profile of single-cell RNA + ATAC in GM-CSF + IL-6 induced MDSCs. **(A)** Illustration of experimental design. **(B)** PCA plot (scRNA-seq) color coded by experimental groups. **(C)** GSEA results for PC1-associated genes. The significant enriched hallmark gene sets (adjusted p value < 0.05) are shown. MDSC-related pathways are highlighted in a red box. **(D)** The motif activity score was computed by chromVAR (scATAC-seq) and subjected to differential testing. Significant differential motifs (adjusted p value < 0.05) between two groups are shown with top 10 over-representative motifs in each group labeled. **(E)** UMAP plot (scRNA-seq) color coded by identified GEX clusters. **(F)** PC1 density plot by GEX clusters. **(G)** Cell composition of GEX clusters across six healthy subjects. **(H)** Gene expression of representative markers in each GEX cluster. **(I)** GSEA results for PC1-associated genes: Significantly enriched UV response-associated gene sets (adjusted p value < 0.05).

To explore the heterogeneity within human M-MDSCs, we utilized a well-established *in vitro* model where MDSCs are generated from PBMCs exposed to GM-CSF + IL-6 (10, 12) (**Figure 1A**). PBMCs from six healthy donors were cultured with or without cytokines. None of the myeloid cells were CD66b+, confirming that all the cells were derived from the monocytic rather than granulocytic lineage (**Supplementary Figure S1A**). Isolated monocytes from the cytokine-treated group (T group) that were CD33+ inhibited T-cell proliferation, whereas CD33+ cells from control (C group) did not (**Supplementary Figure S1B**), confirming that cytokine treatment induced an MDSC phenotype. A bead-enriched CD33+ cell population from both groups was subjected to single-cell multiome analysis. Principle component analysis of the scRNA-seq data demonstrated that the first principal component (PC1) separated the cells from two experimental groups (T group vs. C group) (**Figure 1B**), with the positive side of PC1 (T group) associated with known MDSC-relevant pathways (e.g., glycolysis, mTORC, and ROS production (23, 24), **Figure 1C**). The scATAC-seq data revealed an up-regulation of *AP1* family motifs (*FOS/JUN*), a feature known to be associated with inflammation and immune cell activation (25), and downregulation of *IRF* family motifs after cytokine treatment (**Figure 1D**). *IRF8* downregulation is regarded as another characteristic of MDSCs (8). Taken together, these results confirm that monocytic cells exposed to GM-CSF + IL-6 acquired established MDSC-related transcriptomic, epigenomic, and functional changes.

Next, we used the scRNA-seq data to identify clusters associated with different cell states and referred to these as gene expression (GEX) clusters. This yielded three CD14+ GEX clusters that were distributed along PC1 and representing low, intermediate, and high upregulation of the cytokine-induced MDSC associated transcriptomic profile (CD14+_MDSC_GEXlo, MDSC_GEXint, and MDSC_GEXhi). In addition, two small clusters, one associated with IFN pathway-related genes (CD14+_IFN-specific) and the other CD16+ features (CD14+CD16+ subset), were also identified. The monocytes from the control cells clustered together (CD14+_baseline) and were distinct from the cytokine-treated cells. All the GEX clusters included cells from all six donors, indicating the consistency of the observed patterns. MDSC_GEXhi aligned on the farthest right side of PC1 and represented the profile most consistent with MDSCs, with a high expression of *ITGAM* (CD11b coding gene) and *CEBPB* and a suppressed *IRF8* expression (**Figures 1E–H**, **Supplementary Figure S1C**).

Interestingly, a high number of ultraviolet radiation (UV) response-related gene sets were also significantly associated with PC1 (**Figure 1I**). Genes known to be upregulated after UV exposure were associated with PC1+ and UV-downregulated genes associated with PC1−. This observation indicates that a DNA damage response is closely associated with MDSC generation in this model. As such, we sought evidence for DNA damage using several analytic packages and, surprisingly, using infercnv (26), identified an increase in inferred somatic copy number variation (SCNVs) in the MDSC population. The presence of SCNVs in MDSCs was inferred by comparing the gene expression intensities across genomic positions of cytokine-treated cells to reference cells (control cells) (**Supplementary Figure S2A**). Under “subcluster mode”, we identified two major SCNV clusters. SCNV_hi cells had a large number of inferred SCNVs compared with the SCNV_lo cells (**Supplementary Figure S2B**, **Supplementary Table S1**), and both clusters were identifiable across the six donors (**Supplementary Figure S2C**). Except for the CD14+_baseline cluster, all the other GEX clusters were composed of cells derived from both SCNV_hi and SCNV_lo subsets. MDSC_GEXint and MDSC_GEXhi had a higher ratio of SCNV_hi compared with MDSC_GEXlo (**Supplementary Figure S2D**). By multivariate linear regression, we found that pathways known to be MDSC-related, including glycolysis and mTORC and ROS production, were associated with both gene expression and SCNV profiles (**Supplementary Figure S2E**), whereas the UV response pathways were mostly associated only with the SCNV profile (**Supplementary Figure S2F**). Furthermore, the inferred SCNVs were validated using cyto-SNP microarrays (**Supplementary Figure S3**, **Supplementary Figure S4**). Taken together, these observations provide evidence that a DNA damage response associates closely with the monocytic MDSC formation in this model. The identification of the two SCNV clusters reflects the different DNA damage states among these MDSC clusters.

### Identification and characterization of resistant MDSCs under serum starvation

**Figure 2:**
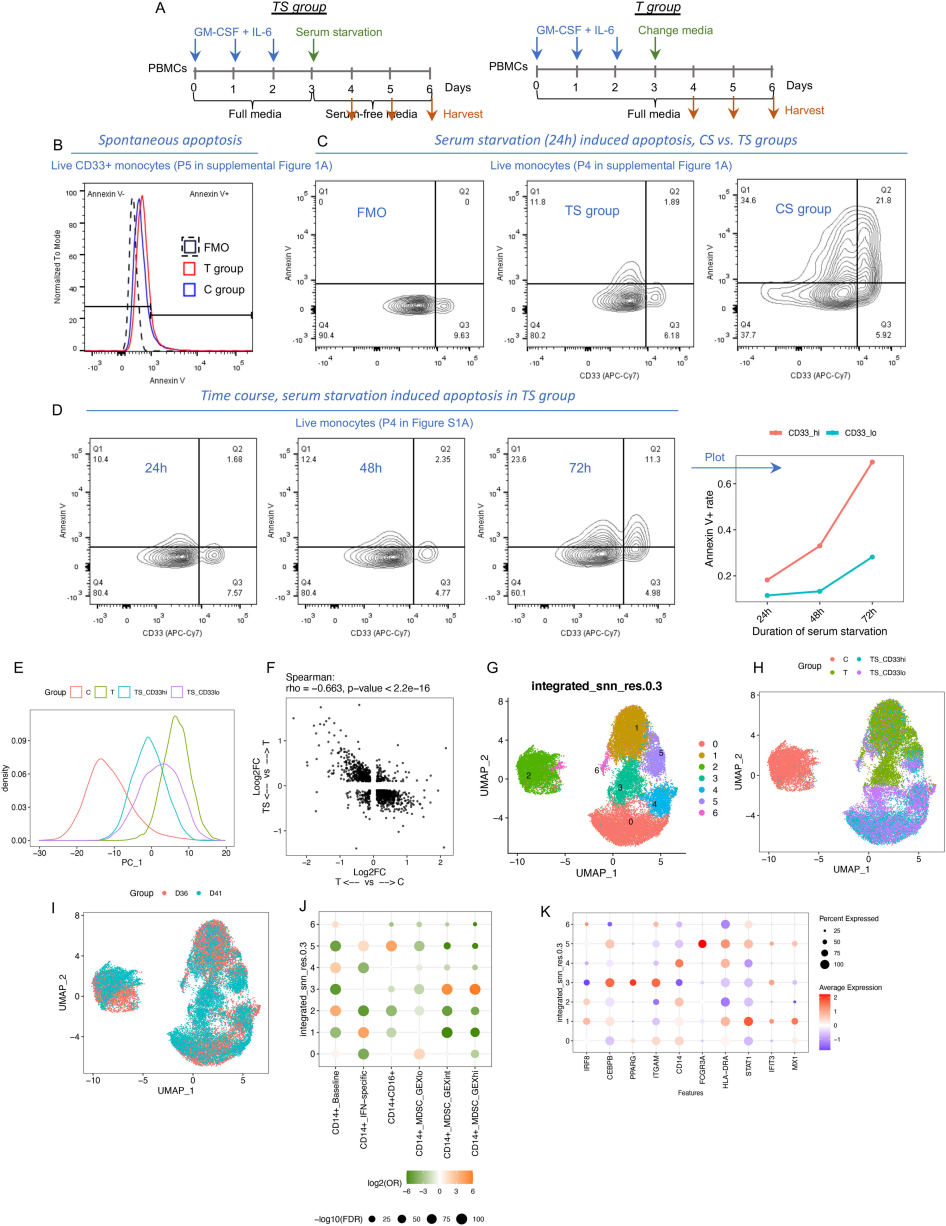
Identification and transcriptomic characterization of resistant MDSCs after serum starvation. **(A)** Experimental design. **(B)** Annexin V staining in the cells cultured in full media. FMO: Fluorescence Minus One control for Annexin V. **(C)** Annexin V staining in the cells cultured after serum starvation for 24 h **(D)** Annexin V staining in cytokine-treated cells after serum starvation for 24, 48, and 72 h **(E–L)** Integrated scRNA-seq data from two different donors (TS group: serum starvation for 48 h). **(E)** PC1 density plot color coded by groups. **(F)** Correlation between the two groups of DEGs. **(G–I)** UMAP plot color coded by GEX clusters identified in this dataset **(G)**, groups **(H)**, and donors **(I)**. **(J)** Enrichment analysis between GEX clusters and the transferred labels using chi-square test. **(K)** Gene expression of representative markers in each GEX cluster.

It is known that harsh microenvironmental conditions can influence the effectiveness of DNA repair pathways (27), and failure of DNA repair can lead to apoptosis (28). MDSCs are often exposed to harsh conditions such as the depletion of nutrients as encountered in the tumor microenvironment. Our observation that sustained inflammatory cytokine exposure leads to DNA structural changes that were tolerated by newly formed MDSCs led us to postulate that additional cell stress might further select for MDSC more representative of MDSC encountered *in vivo*. As such, we adopted serum starvation as a common cell culture approach to induce cellular stress (29, 30). PBMCs from C and T groups were cultured without serum (labelled as CS and TS groups). The apoptotic rate (a consequence of cellular stress response) was evaluated within monocytic cells and defined as the ratio of Annexin V+ (Ap+) cells to Ap− cells. The spontaneous apoptosis rate in complete media that includes serum was ~10% in the C and T groups. Serum starvation for 24 h dramatically induced apoptosis in monocytes from the CS group (~50% Ap+), whereas the counterpart from the TS group was resistant to apoptosis (~10% Ap+). Interestingly, serum starvation yielded two distinct populations of monocytic cells in the TS group including a CD33^lo^ subset, as assessed by flow cytometry, that displayed a lower apoptotic rate than a CD33^hi^ subset. The differences in apoptotic rates continued to diverge between these two subsets with serum starvation for 48 and 72 h (**Figures 2A–D**).

To characterize the gene expression profile of the two CD33 subsets, we flow sorted CD33^hi^ and CD33^lo^ cells from the TS group (serum starvation for 48 h) as well as CD33+ cells from the C and T groups (cultured with serum) from two different donors (D36 and D41) and performed scRNA-seq. As shown in **Figure 2E**, PC1 represents the cytokine-induced changes. Serum starvation partially reversed these MDSC-related changes as shown by comparing cells from the TS group to those from the T group (**Figures 2E, F**). We identified seven GEX clusters (C0-C6) across the C, T, and TS groups (**Figures 2G–L**, **Supplementary Figure S5**). C6 cells were relatively low quality with high mitochondrial genes. The other clusters included C2: mainly from the C group; C5: CD14+CD16+; C1: mainly from the T group; C0 and C4: mainly from the TS group; and C3: identifiable from both T and TS groups. Of all the clusters, only C3 was consistently higher in CD33^lo^ cells compared with CD33^hi^ cells across two donors (**Supplementary Figure S5F**). Label transfer analysis using the scRNA-seq data shown in **Figure 1** as reference showed that only C3 was significantly enriched in MDSC_GEXhi and MDSC_GEXint, whereas other clusters were enriched in MDSC_GEXlo or baseline transcriptomic patterns (**Supplementary Figure S5F**). Thus, the C3 cluster had the strongest MDSC-related profile and was also identifiable under serum starvation.

**Figure 3:**
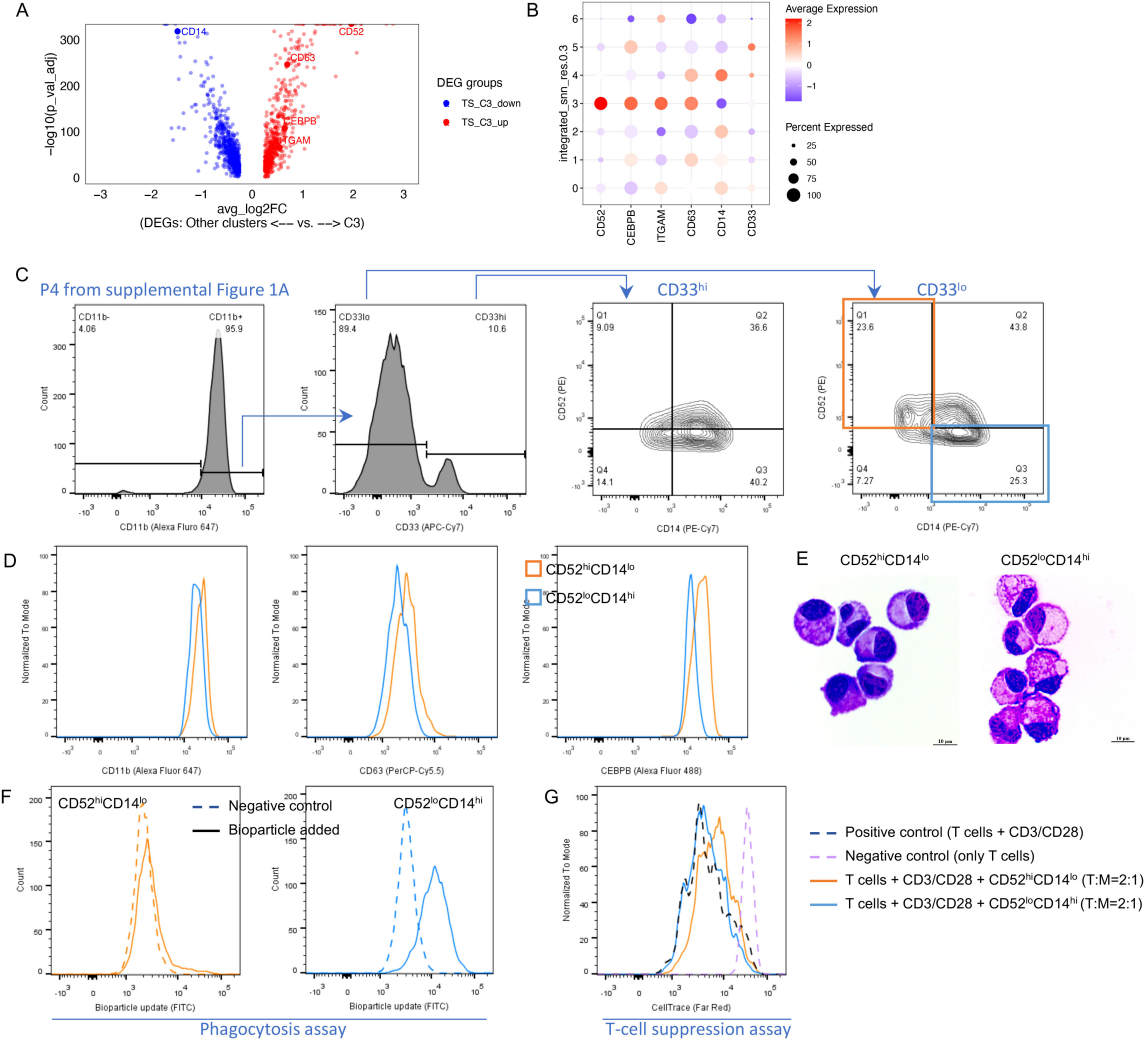
Transcriptional and functional characterization of resistant MDSCs in the TS group. **(A)** Significant DEGs between C3 and other clusters in the TS group. **(B)** Gene expression of representative markers in each GEX cluster. **(C–G)** Phenotypic and functional validation of resistant MDSCs (using the cells from the TS group under serum starvation for 48 h). **(C)** Flow cytometry validated the enrichment of resistant MDSCs (CD52hiCD14lo) in CD33lo compared with CD33hi after serum starvation (upper left quadrants, third vs. fourth panels). **(D)** Flow cytometry validated the other markers for resistant MDSCs. **(E)** Diff-Quik Staining for flow sorted populations. **(F)** Results of the phagocytosis assay. **(G)** Results of the T-cell suppression assay.

Among the top DEGs upregulated in C3 was the cell surface marker *CD52*, whereas *CD14* was markedly downregulated (**Figures 3A, B**). We used these markers to attempt to identify the C3 cluster by flow cytometry and observed that CD52^hi^CD14^lo^ cells were dominant in CD33^lo^ cells (23.6% in CD33^lo^, 4^th^ panel of **Figure 3C**) compared with CD33^hi^ cells (9.09% in CD33^hi^, 3^rd^ panel of **Figure 3C**). CD52^hi^CD14^lo^ cells expressed higher CD11b, CD63, and C/EBP-beta compared with CD52^lo^CD14^hi^ cells. (**Figure 3D**). Thus, we were able to demonstrate the presence of cell cluster C3 after serum starvation by flow cytometry across multiple donors. Next, we flow sorted CD52^hi^CD14^lo^ and CD52^lo^CD14^hi^ populations. Both exhibited a mature monocyte morphology with dense nuclei (**Figure 3E**). However, CD52^hi^CD14^lo^ cells more potently suppressed T-cell proliferation and exhibited almost no capacity for bioparticle uptake compared with CD52^lo^CD14^hi^ cells, two functional characteristics of MDSCs (**Figures 3F, G**). Thus, we were able to identify and validate an MDSC cell subset (cluster C3) that was resistant to the harsh conditions created by serum starvation. In the remainder of the paper, we refer to the cells identified in cluster C3 (further defined as CD52^hi^CD14^lo^) as resistant MDSCs (rMDSCs).

Next, we sought to determine how these clusters associated with different DNA-damaged states. SCNVs were inferred in the GEX clusters C3, C0, and C4 (from TS group) using the monocytes from the C group as reference (**Supplementary Figure S6**). C3 (rMDSC) and C4 both had a high number of SCNVs, in contrast to the limited SCNVs in C0. Both C4 and C0 were less MDSC-like compared with C3. Thus, the gain of SCNVs was not unique to the formation of rMDSCs. However, these findings do demonstrate that rMDSCs were able to maintain their immune suppressive features under cellular stress created by serum starvation, despite the presence of high DNA damage. We reasoned that these rMDSCs should have unique features relevant to their resistance. These features should render the cells more stable and less susceptible to the changes in their microenvironment. This could contribute to the resilience of MDSCs commonly encountered in inflammatory conditions and tumors. Thus, we hypothesized that features preserved in rMDSCs may yield a signature highly representative of monocytic MDSCs.

### Identification and validation of a stress-tolerant gene coexpression module as a common MDSC signature

**Figure 4:**
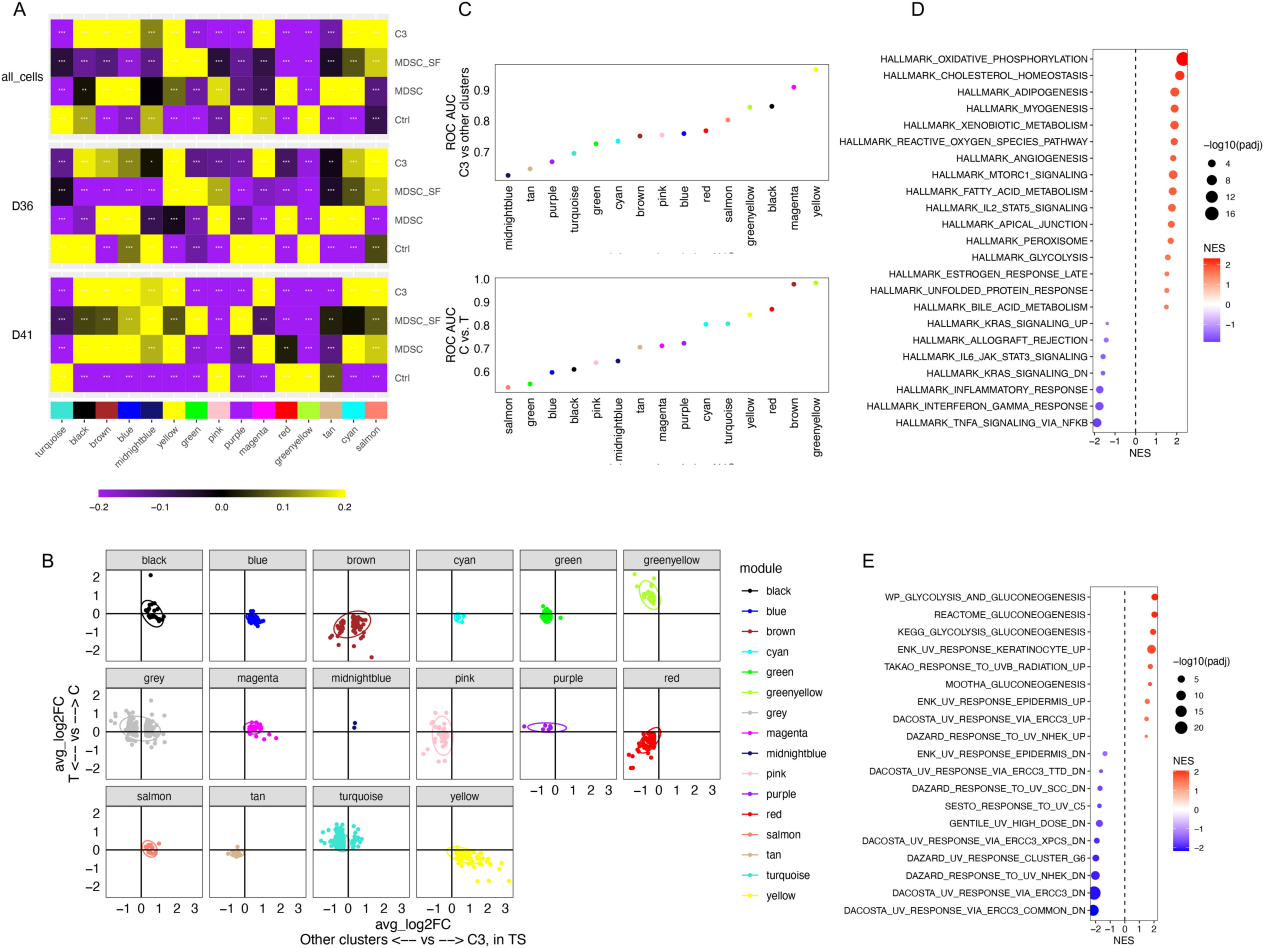
Identification of a microenvironment-tolerant gene coexpression module highly expressed in rMDSCs. **(A)** Gene coexpression modules were identified using hdWGCNA. Module-trait correlations were shown (Ctrl: C group; MDSC: T group; MDSC_SF: TS group; C3: cell cluster 3 characterized in **Figure 1G**). **(B)** DEGs were identified between C and T groups (shown in y-axis) and between the rMDSCs (C3) and other cells in the TS groups (shown in x-axis). The shared DEGs were plotted using log2 fold-change (FC) in each comparison. The genes were colored based on the gene coexpression modules. **(C)** The ability to distinguish between different groups of cells was evaluated using the signature scores for each module. Data were fitted using logistic regression. The area under the curve (AUC) of ROC was computed. **(D, E)** GSEA results using the genes included in hdWGCNA analysis ranked by kME value for the yellow module. **(D)** Significant enriched hallmark gene sets. **(E)** The significant enriched curated gene sets related to UV response and gluconeogenesis.

Gene coexpression networks are closely related to key biological processes or mechanisms. The hdWGCNA package (18) was used to identify gene co-expression modules across the C, T, and TS groups (**Figure 4A**, **Supplementary Table S2**). To prioritize the top rMDSC-specific modules, we aimed to select the modules that were both MDSC-related (induced by cytokine exposure) and stress-tolerant (preserved under serum starvation). Therefore, gene modules that contained genes from the two groups of DEGs, one reflecting the differences between the C and T groups (shown in the y-axis in **Figure 4B**) and the other representing the differences between the rMDSCs and other cells in the TS group (shown in the x-axis in **Figure 4B**), were down-selected. This narrowed the gene modules of interest to the three shown in **Figure 4B** labeled in yellow, blue, or cyan and depicted in the right lower quadrant of the graph. Among these three modules, the signature score derived from the yellow module was found to achieve the best separation between the two groups of cells in both comparisons (**Figure 4C**), and this module was selected for further analysis.

To interpret the biological meaning of the yellow module, GSEA was performed using the genes included in hdWGCNA analysis based on the kME (module eigengenes) value for the yellow module as the rank. As expected, the yellow module was positively associated with MDSC-related pathways (e.g., mTORC and ROS production (23, 24)) and the *CEBPB* regulon, whereas the *IRF8* regulon was enriched in the negative side (**Figure 4D**, **Supplementary Figure S7**). The yellow module was also associated with a high DNA damage response and metabolic pathways including gluconeogenesis (**Figure 4E**).

**Figure 5:**
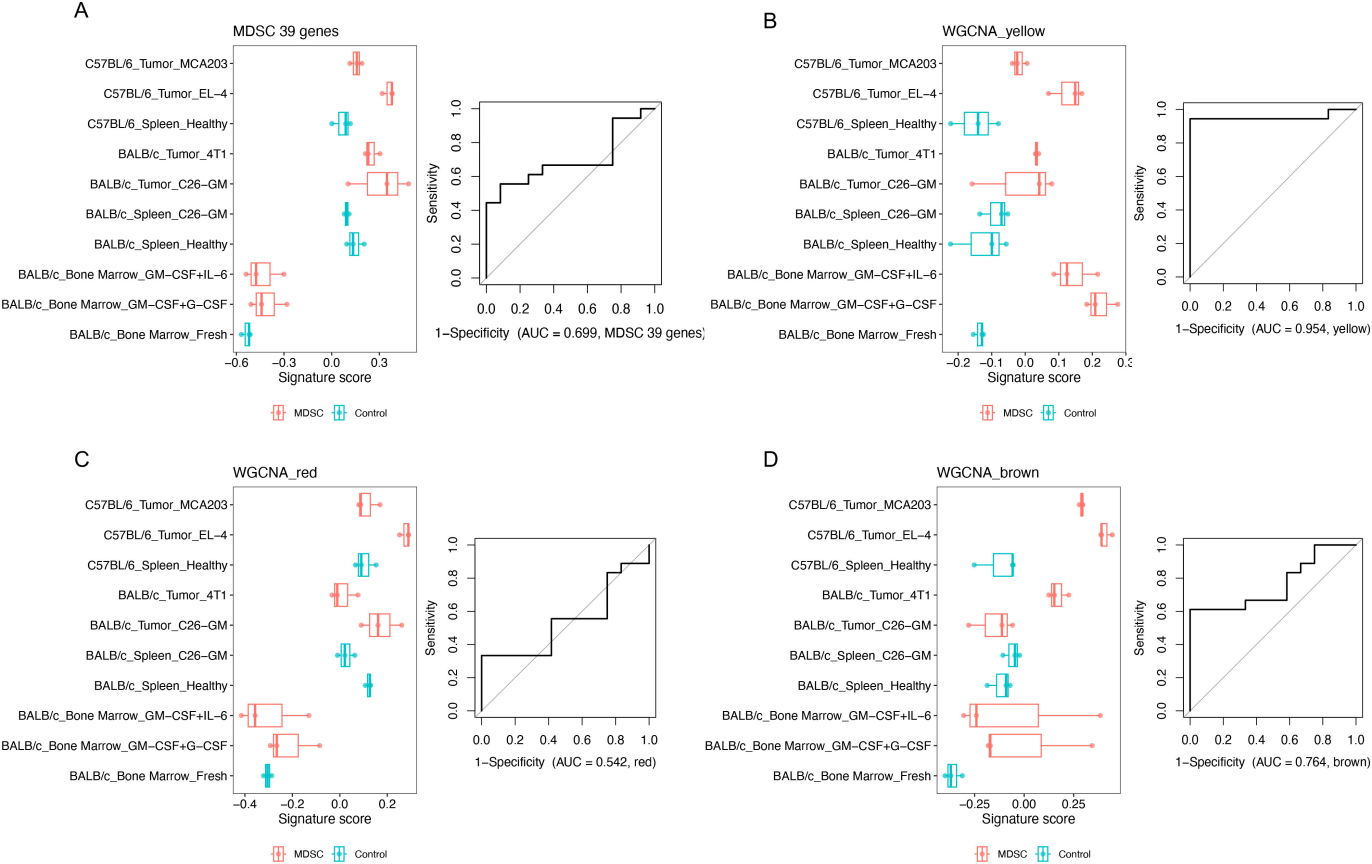
Demonstration of the yellow module as a common MDSC signature across different sources. A published MDSC dataset GSE21927 was analyzed. For each gene set or module, the signature score was calculated for each sample. Logistic regression was fitted between signature scores and categories (MDSC or control) across all the samples. ROC AUC was shown to evaluate how this group of genes can distinguish MDSCs from control cells. **(A)** Public MDSC signature. **(B–D)** The gene coexpression MDSC-related modules computed in our study, with different compositions of microenvironment-tolerant vs. sensitive genes: **(B)** Yellow module (mostly microenvironment-tolerant genes). **(C)** Red module (mostly microenvironment-sensitive genes). **(D)** Brown module (half tolerant + half sensitive genes).

Next, to determine if the yellow module is also upregulated in other MDSCs, we utilized a bulk transcriptomic dataset that included multiple sources of MDSCs (19). This dataset encompassed both *in vitro* models of cytokine-induced MDSCs and *in vivo* MDSC models from tumor-bearing mice. We also compared the yellow module signature with a well-accepted human MDSC signature (31). This published 39-gene signature was indeed expressed higher when comparing MDSCs with the corresponding control cells from a single source (**Figure 5A**, left panel), supporting the effectiveness of this signature. However, this was not always the case when comparing the signatures between different sources of MDSCs. This resulted in an ROC AUC of 0.699 for distinguishing MDSCs from non-MDSCs across different sources (**Figure 5A**, right panel). In contrast, the gene signature score of the yellow module genes performed well in separating MDSCs from the control cells regardless of the sources (ROC AUC = 0.954, **Figure 5B**). As an additional comparison, we assessed the ROC curve for MDSC of the red and brown modules from our analysis. Both are the gene modules upregulated in the MDSCs we generated *in vitro* (**Figure 4B**). The red module, mostly containing genes impacted by serum starvation, showed a poor separation for MDSCs (ROC AUC = 0.542, **Figure 5C**). The brown module, containing about half serum starvation tolerant genes and half sensitive genes, showed a better separation (ROC AUC = 0.764, **Figure 5D**) than the red module, but still not as strong as the yellow module. This analysis demonstrates that among the MDSC-related gene modules, the gene signature associated with tolerance to cellular stress achieved a better consistency across different sources of MDSCs than the genes associated with sensitivity to serum starvation. Of the genes coding for conventional MDSC surface markers, *ITGAM* (CD11b coding gene) was included in the yellow module, whereas *CD14*, *CD33*, *S100A8*, and *S100A9* were not (**Supplementary Table S2**). This highlights CD11b as a better marker for MDSCs based on resistance to cellular stress. *CD52*, a marker associated with rMDSCs was also included in the yellow module. Thus, we were able to show that the elevated yellow module genes are a common feature across multiple monocytic MDSC populations using an independent dataset.

### Identification of rMDSC-like cells in the tumor microenvironment of human cancers

**Figure 6:**
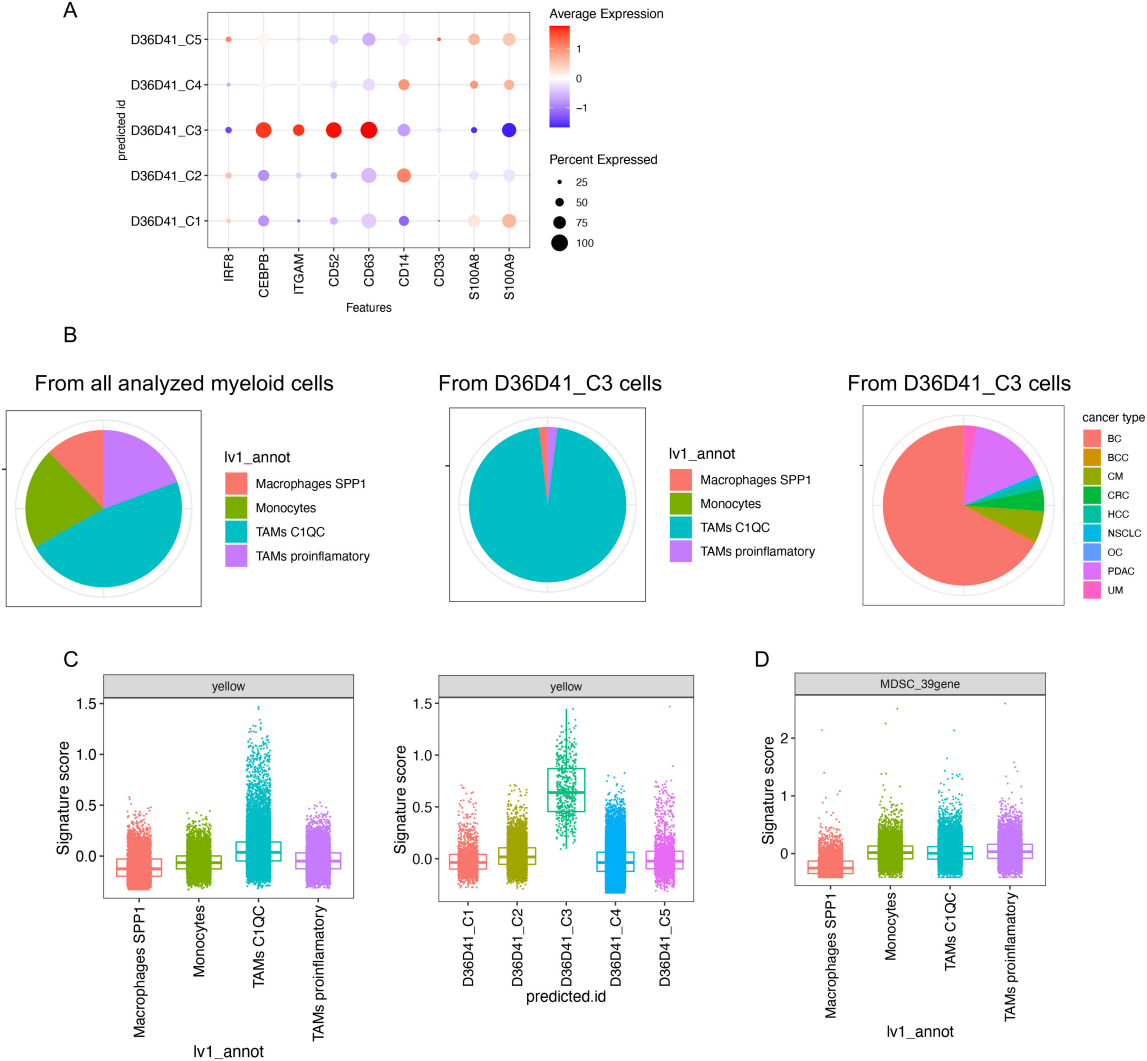
Identification of rMDSC-like cells in the tumor microenvironment of human cancers. A published scRNA-seq dataset integrating the tumor microenvironment across different cancer types (TICAtlas) was analyzed. **(A)** Gene expression of representative markers in each predicted cell label using our scRNA-seq data shown in **Figure 1G** as a reference. **(B)** Cell composition is shown in pie charts. **(C)** Signature scores of the yellow module grouped by the annotations provided by the original dataset (left) or by the predicted cell labels (right). **(D)** Signature scores of the published 39-gene MDSC signature grouped by the annotations provided by the original dataset. BC, breast cancer; BCC, basal cell and squamous cell carcinomas; CM, cutaneous melanomas; CRC, colorectal cancers; HCC, hepatocellular carcinomas; NSCLC, non-small-cell lung cancers; OC, ovarian cancers; PDAC, pancreatic ductal adenocarcinomas; UM, uveal melanomas.

We next sought evidence for the presence of rMDSCs-like cells in human tumors by querying a published scRNA-seq dataset from the tumor immune atlas. This data resource integrated published datasets from 13 cancer types derived from 217 patients (20). This dataset is ideal to computationally scan for the presence of rMDSC-like cells in the tumor microenvironment *in vivo*. We extracted the cells in the monocyte–macrophage lineage and excluded the proliferating cells since they are a mixed population that includes both monocytes and macrophages (**Figure 6**, **Supplementary Figure S8**, **Supplementary Table S3**). Using our scRNA-seq dataset as a reference, we annotated the selected cells using label transfer. We were able to identify the cells predicted to be rMDSCs in 9 out of 13 cancer types. The predicted rMDSCs were also associated with some conventional MDSC markers, including high *ITGAM* and *CEBPB* and low *IRF8* (**Figures 6A, B**).

C1Q+ tumor associated macrophages (C1Q TAMs) have been correlated with poor outcome (32) and colocalize with exhausted T cells often in the area in fibrotic tissue (20). Overall, the C1Q TAMs annotated by the original dataset express a higher level of yellow module compared with other subtypes of macrophages or monocytes (**Figure 6C**, left panel). This demonstrates that the yellow module signature was dominant in a cell population known to be immunosuppressive. Interestingly, most of the cells predicted to be rMDSCs corresponded to C1Q TAMs that express extremely high yellow module genes (**Figure 6C**, right panel; **Figure 6B**, middle panel). We speculate that C1Q TAM may have a gene expression background that favors the high expression of the yellow module signature, and this background may favor the formation of rMDSCs. In contrast, 39-gene signature was not highly expressed in C1Q TAM (**Figure 6D**), indicating that the 39-gene signature cannot be used to explain the immunosuppressive association reported in C1Q TAM.

### The independent prognostic value of the yellow module signature in AML patients

**Figure 7:**
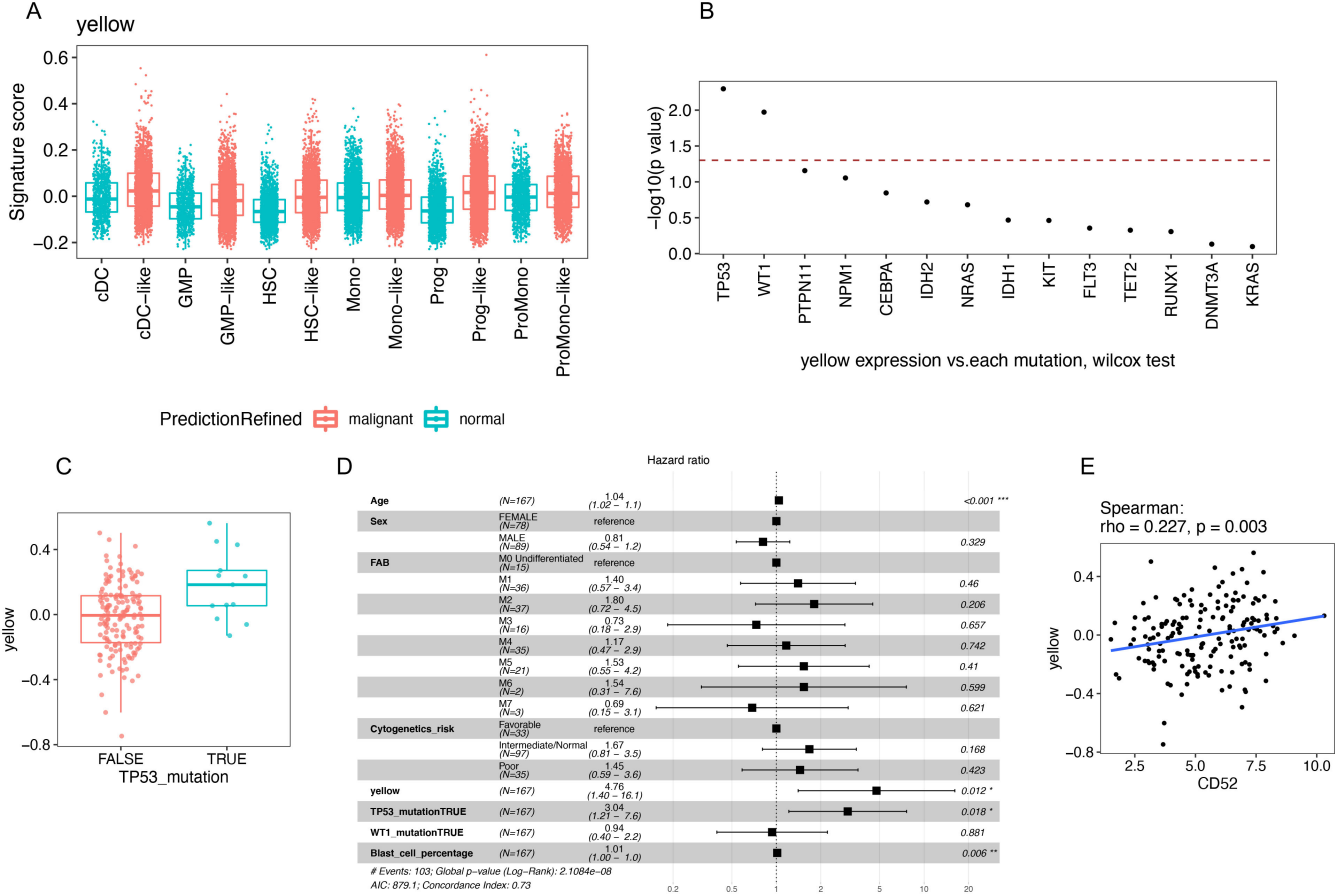
Identification and the independent prognostic value of the yellow module signature in AML patients. **(A)** Signature score of the yellow module was calculated in myeloid and progenitor cells using a published AML scRNA-seq dataset (GSE116256). **(B–E)** Analysis of the TCGA AML dataset. **(B)** Wilcox test was evaluated between each mutation and the yellow module signature score. Mutations were sorted by −log10(p value). Dashed line annotated where p value = 0.05. **(C)** TP53 mutation was significantly associated with higher yellow module signature score. **(D)** Survival analysis using the Cox regression model. **(E)** Correlation between CD52 gene expression and the yellow module signature score.

A feature of cancer cells is the gain of resistance to cell death leading to a survival advantage over non-cancer cells [25]. Because the yellow module was also associated with the resistance of myeloid cell apoptosis, we wondered whether the yellow module could be identified in AML. We queried an scRNA-seq AML dataset (33) and found that no myeloid cells in this dataset were predicted to be rMDSCs. However, the malignant myeloid cells and their progenitors expressed an increase in the yellow module signature compared with the corresponding normal cells (**Figure 7A**). Therefore, rMDSCs are clearly distinct from malignant myeloid cells; however, genes that comprise the yellow module co-expression network may play a role in myeloid cancer cells.

We next queried the TCGA AML dataset to examine the association between the yellow module signature and clinical outcomes (**Figures 7B–E**, **Supplementary Figure S9**). We constructed a multivariate cox regression model that included the yellow module signature, age, sex, FAB classification, known AML prognostic factors (cytogenetic risk category and blast percentage), and mutations that are significantly associated with yellow module (*TP53* and *WT1*) (**Figure 7B**). *TP53* is a known tumor-suppressor gene that can be activated by DNA damage and induce cell cycle arrest or apoptosis (34). In the TCGA AML dataset, mutation of *TP53* was significantly associated with a high level of yellow module expression (**Figure 7C**). Consistent with the original paper, *TP53* mutation was a strong predictor of poor outcome and the only significant mutation for poor prognosis in multivariate analysis (21). Strikingly, a time-to-event analysis revealed that the yellow module signature was independently associated with poor prognosis in AML beyond the known prognostic markers, including mutated *TP53* (**Figure 7D**). The independent prognostic value may be due to the survival advantage associated with the yellow module that cannot be fully explained by the gain of *TP53* mutations.

High expression of *CD52* has been reported to be associated with poor prognosis in several subtypes of AML, including normal karyotype (35), high *EVI1* (36), and *FLT3-ITD* mutated AML (37). In our network analysis in **Figure 4**; **Supplementary Table S2**, *CD52* was among the top 10 hub genes in the yellow module. Therefore, we assessed the correlation between the expression of *CD52* and the yellow module signature in AML patients. In the TCGA data analysis, the yellow module signature was significantly and positively correlated with *CD52* (spearman correlation: rho = 0.227, p = 0.003, **Figure 7E**). Furthermore, the yellow module signature showed a more significant prognostic value than *CD52* gene expression in the presence of *TP53* mutation (**Supplementary Figure S9**). These findings suggest that upregulation of the yellow module may be biologically manifested as poor prognosis in *CD52* hi AML patients.

## Discussion

The goal of this study was to deconvolute the heterogeneity of M-MDSCs and identify common features conserved across different sources of M-MDSC populations. We accomplished this by inducing M-MDSCs under stress conditions, reasoning that MDSC often arise and survive in harsh microenvironments that include sustained inflammation and nutrient or growth factor depletion. The culture conditions of continuous pro-inflammatory cytokine exposure and serum starvation generated a myeloid cell population that exhibited functional features of MDSCs. A transcriptomic signature representing the combined effects of inflammatory cytokine exposure and cell stress through serum starvation had a high ROC AUC for the separation of MDSCs from control cells across different sources. This signature was also upregulated in C1Q macrophages (known to be associated with immunosuppression) in the tumor microenvironment from multiple cancer types. In addition, the signature was upregulated in AML cells and was independently associated with poor survival in the TCGA AML dataset. Our study highlights the role of genes reflecting tolerance to cellular stress in distinguishing MDSCs from the conventional myeloid cells.

The DNA damage response identified within cytokine-induced MDSCs suggested that cell stress responses could be a feature contributing to MDSC heterogeneity. We further reasoned that MDSCs often either arise in and/or survive in microenvironments that have not only inflammatory signals but also other cell stressors that might induce adaptive pathways that could apply selection pressure for MDSC survival. This concept was supported by the enrichment of a CD11b+CD52hiCD14lo cell subset in the CD33lo cell population that was resistant to apoptosis. The selective upregulation of genes involved in metabolism and the DNA damage response in these rMDSCs likely confers a survival advantage. This conclusion is supported by the identification of rMDSCs or the upregulation of the yellow module signature in cells in the tumor microenvironment from different cancers and in malignant myeloid cells from AML patients. It is also notable that serum starvation partially reversed the MDSC features induced by cytokine treatment alone, raising the possibility that the early cell state in the pathway to transition to myeloid MDSC formation could be reversed by certain cell stressors. Thus, the cell stress features found within harsh microenvironments high in MDSC numbers (i.e., tumor microenvironments and sustained inflammation) could select for MDSCs with a survival advantage.

Our analysis of the yellow module signature in AML demonstrated that the signature was associated with mutated *TP53* and was independently associated with poor prognosis. This association was independent of the known predictors of outcomes (e.g., *TP53* mutations, cytogenetic alterations, blast cell percentage). This analysis supports the possibility that high expression of the yellow module signature contributes to an autonomous state necessary for maintaining survival of immune suppressive myeloid cells. This analysis also highlights the potential importance of the gene network represented by the yellow module in AML. Currently, the study of MDSC in AML are very limited (38). Establishing whether *CD52*hi cells in AML patients also have immunosuppressive function requires further investigation.

Our study also has limitations. We used serum starvation as a cell stressor. This does not necessarily replicate *in vivo* conditions such as those likely encountered in the tumor microenvironment. Since the gene signatures induced by serum starvation *in vitro* were easily detected in MDSCs *in vivo*, we reason that there are responses that are common across multiple cell stressors. We also acknowledge that we utilized computational methods to establish the presence of rMDSC-like cells in tumor microenvironments, using preexisting scRNA-seq datasets. To fully characterize rMDSC-like cells within a specific cancer type will require further investigation.

In summary, we identify and characterize a subset of MDSC (rMDSCs) resistant to cellular stress. The scRNA-seq dataset used to identify rMDSC will serve as an excellent reference dataset to identify rMDSC-like cells in scRNA-seq datasets from human disease states. We also reveal a co-expression set of genes (the yellow module) as a common feature shared across different sources of MDSCs. The gene signature derived from the yellow module can be used to detect M-MDSC cells *in vitro* and *in vivo* in bulk or single-cell transcriptomic datasets. Further study on the effect of microenvironmental factors on MDSCs may lead to a more refined molecular classification of MDSCs.

## Data Availability

The datasets presented in this study can be found in online repositories. The names of the repository/repositories and accession number(s) can be found below: GSE248954, GSE249243 (GEO).

## Supplementary material

Supplementary Table S1 Infercnv cell annotation.

Supplementary Table S2 WGCNA consensus modules.

Supplementary Table S3 Immune altas cell annotation.
