# Supplementary material for: Single-cell transcriptomic analysis identified resistant MDSCs and a stress-tolerant gene co-expression network as common MDSC features across multiple disease settings
Source: Front Immunol. 2025 Apr 8;16:1565211. doi: 10.3389/fimmu.2025.1565211 (PMC12011849; doi:10.3389/fimmu.2025.1565211)
Supplement: Supplementary file 4 [file DataSheet1.docx]

**SUPPLEMENTAL FIGURES AND FIGURE LEGENDS**

**
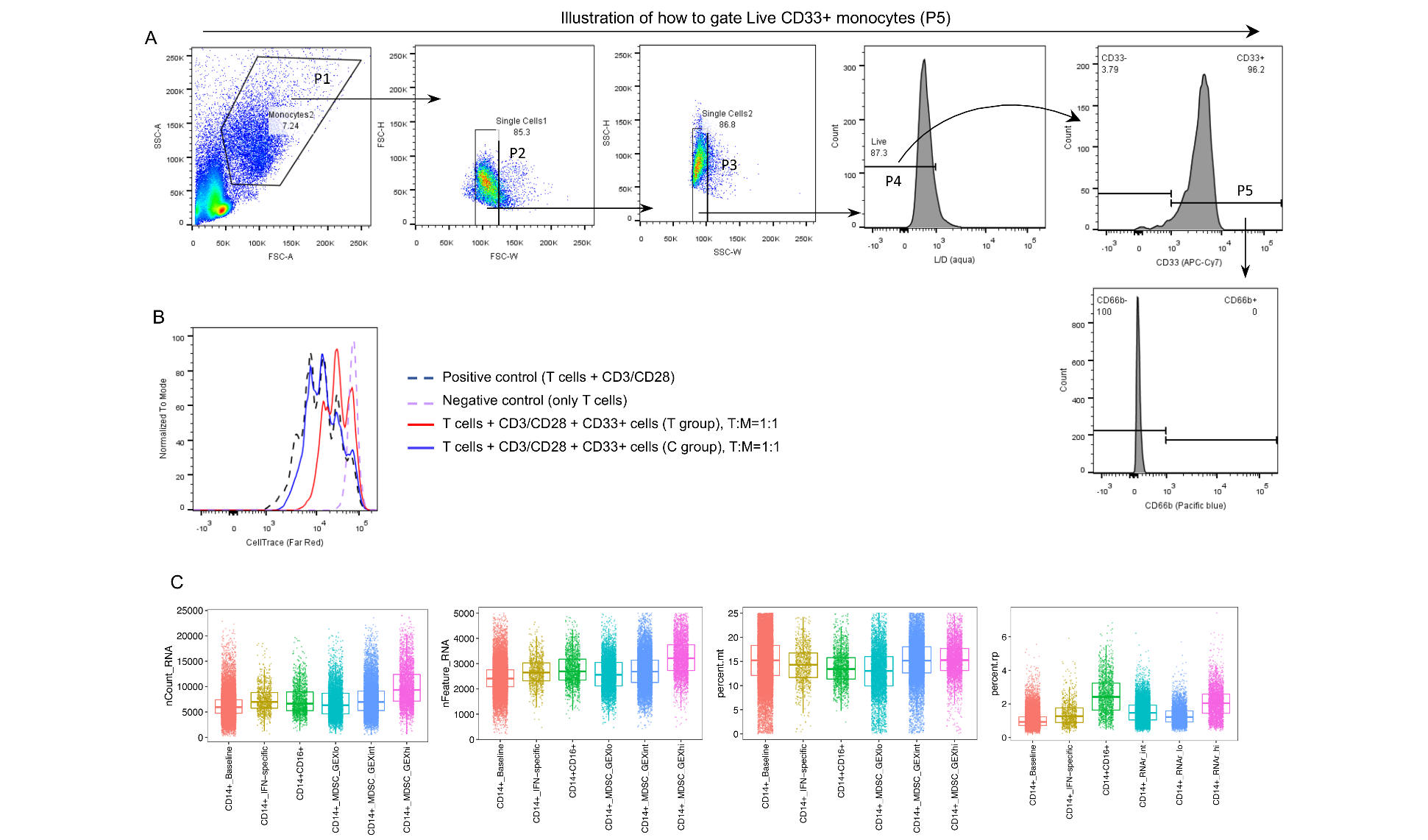
**

**Fig. S1. Supplemental data for Fig. 1**. (A) Illustration of how gating was performed for live CD33+ monocytes. (B) T cell suppression assay. T cells were stained with CellTrace Far Red dye. Flow sorted CD33+ monocytes from C or T group were added to T cells in M:T = 1:1. Human CD3/CD28 dynabeads were added at a bead-to-cell ratio of 1:1. For each donor, duplicates were used for each experimental condition. Cells were co-cultured for 4 days, and then harvested, stained for anti-human CD3 SuperBright 600 and live/dead dye, and evaluated by flow cytometry. (C) Parameters of quality control across GEX clusters.


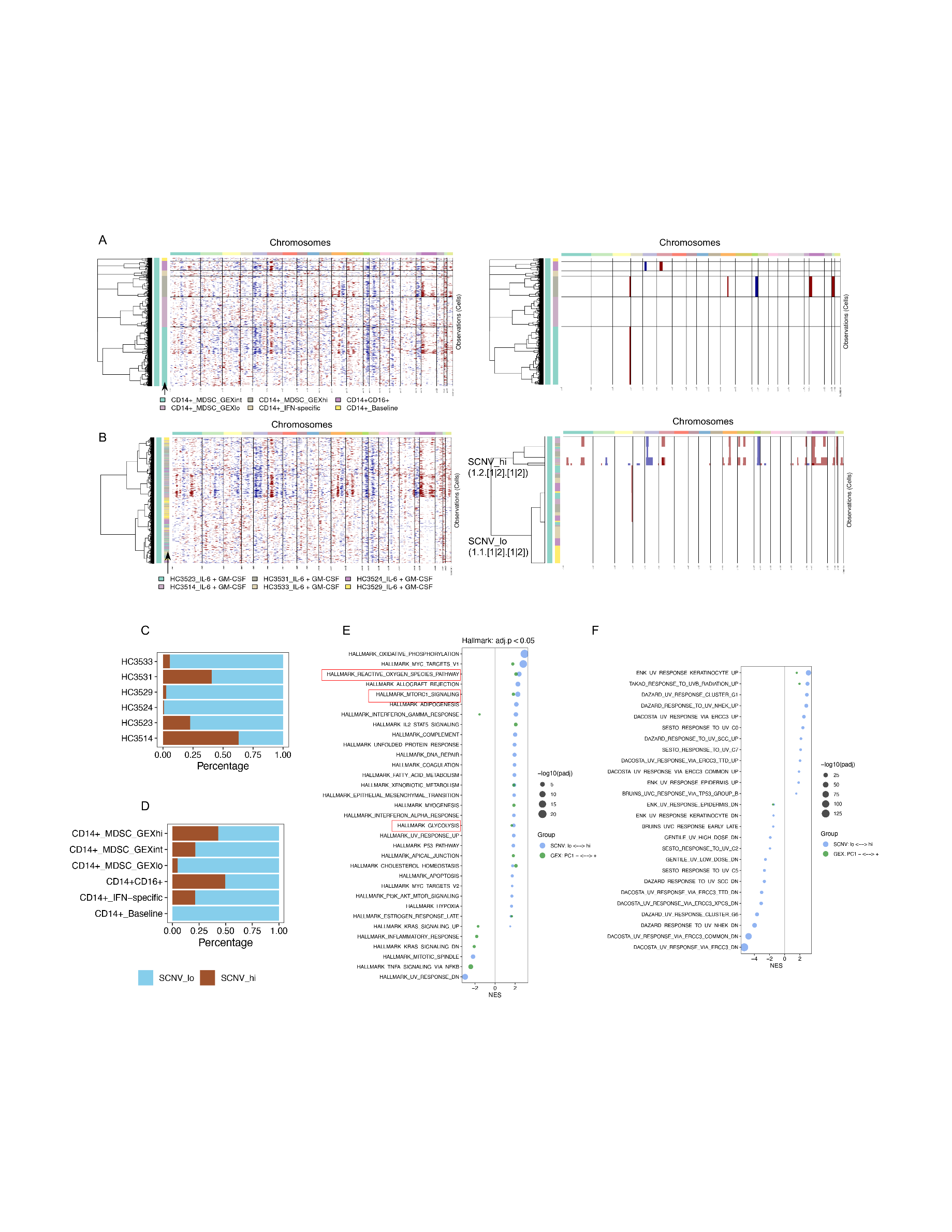


**Fig. S2. Inferred SCNV profile in GM-CSF + IL-6 induced MDSCs using scRNA-seq data.** (A-B) Left panel: The expression values for the genes ordered by genomic locations across the cells from T group (red: gain vs. blue: loss) using the cells from C group as reference. Right panel: Predicted SCNVs using 6-state Hidden Markov Model (HMM). (A) SCNVs were inferred as average within each GEX cluster. (B) SCNVs were inferred under “subcluster” mode regardless of the GEX cluster. (C-D) The composition of the two SCNV clusters among the cells from T group, grouped by (C) donors or (D) GEX clusters. (E-F) To deconvolute the transcriptomic changes associated with SCNVs, we performed multivariate linear regression. PC1 coordinates (shown in Fig. 1B, labeled as GEX_PC1), SCNV clusters, subject IDs and cell cycle phases were selected as four independent variables. To biologically interpret the gene expression that can be explained by SCNVs or not, we ranked the significant genes based on the t values of SCNV clusters or GEX_PC1 and performed GSEA separately. Significantly enriched gene sets associated with GEX_PC1 or SCNV clusters were shown: (E) Hallmark gene sets, and (F) UV response related gene sets.


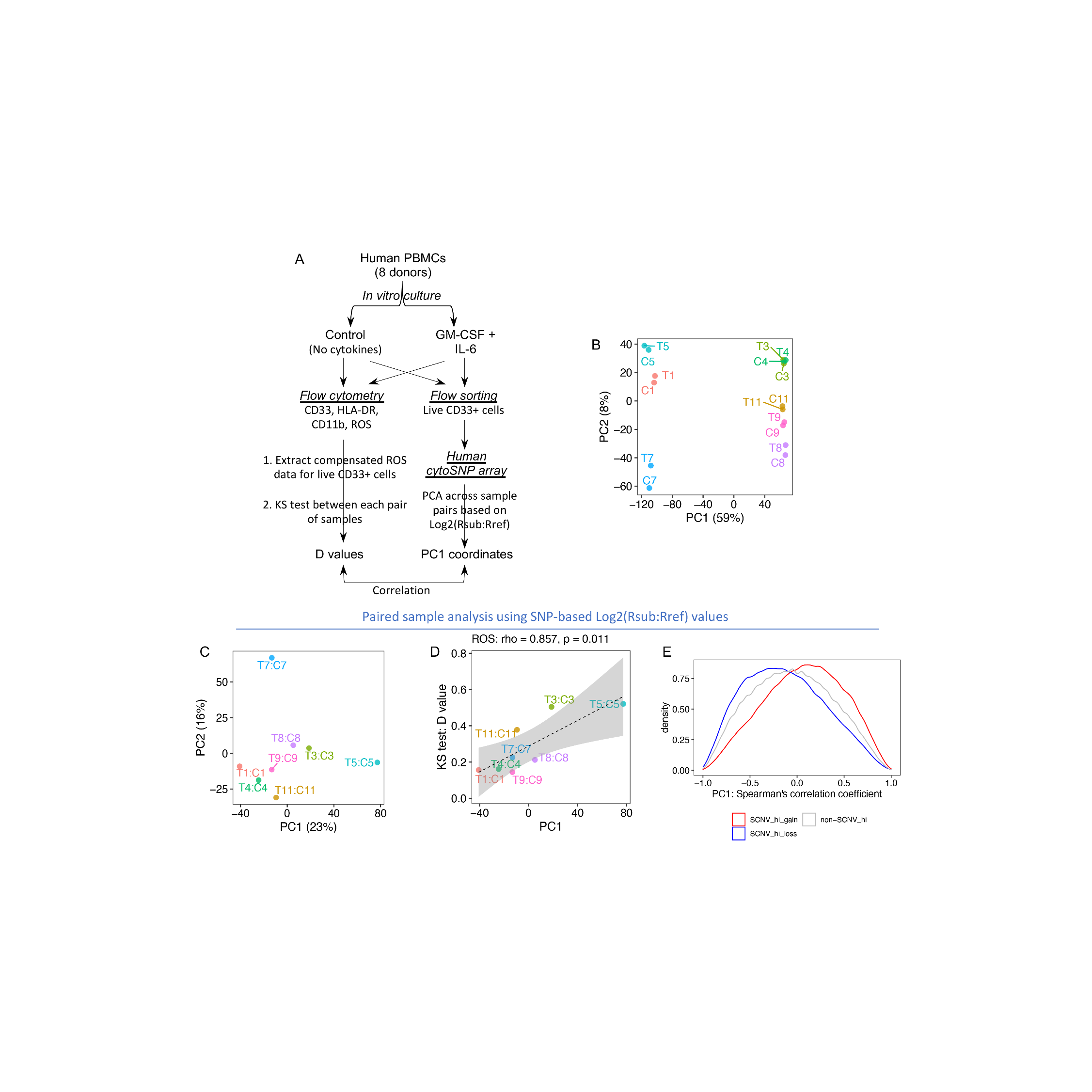


**Fig. S3: Validation of inferred SCNVs using human cyto-SNP array.** (A) Schematic of the experimental and analytic workflow. (B) PCA plot based on Log2R values across 16 samples. (C-E) Analysis using a Paired Sample Table. (C) PCA plot based on Log2(Rsub:Rref) values across 8 pairs. (D) The magnitude of ROS shift is significantly correlated with the PC1 coordinates shown in (C). Spearman’s correlation coefficient and p value are shown. (E) Distinct distributions of correlation coefficients with PC1 coordinates shown in (C) across SNPs under three different categories.


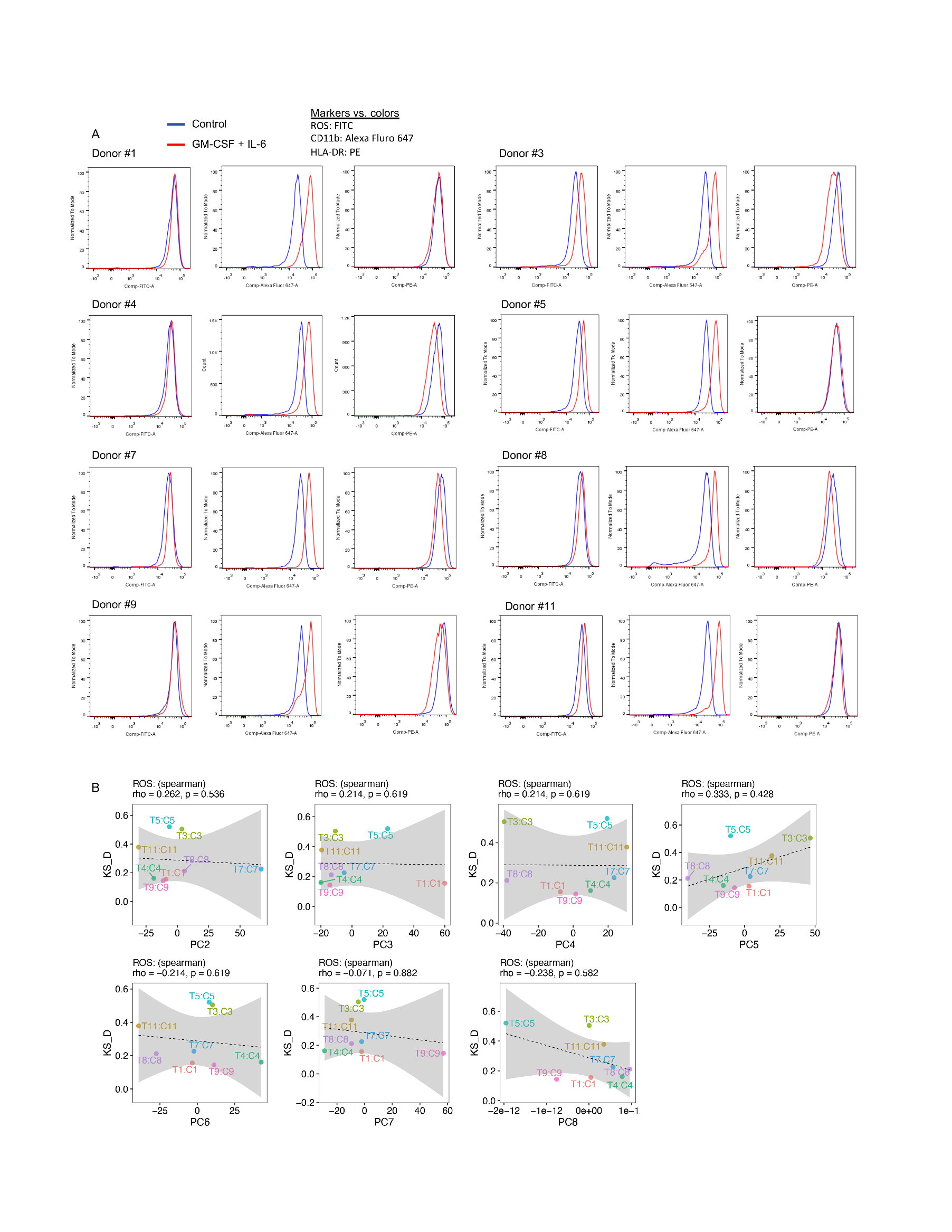


**Fig. S4. Additional data for Fig. S3.** (A) Changes in surface markers (CD11b, HLA-DR) and cellular ROS in live CD33+ cells from 8 donors between two experimental groups (16 samples subjected to human cytoSNP array). Results are shown by each donor. (B) Correlation between ROS shift (D value from KS test) and the coordinates of other 7 PCs.


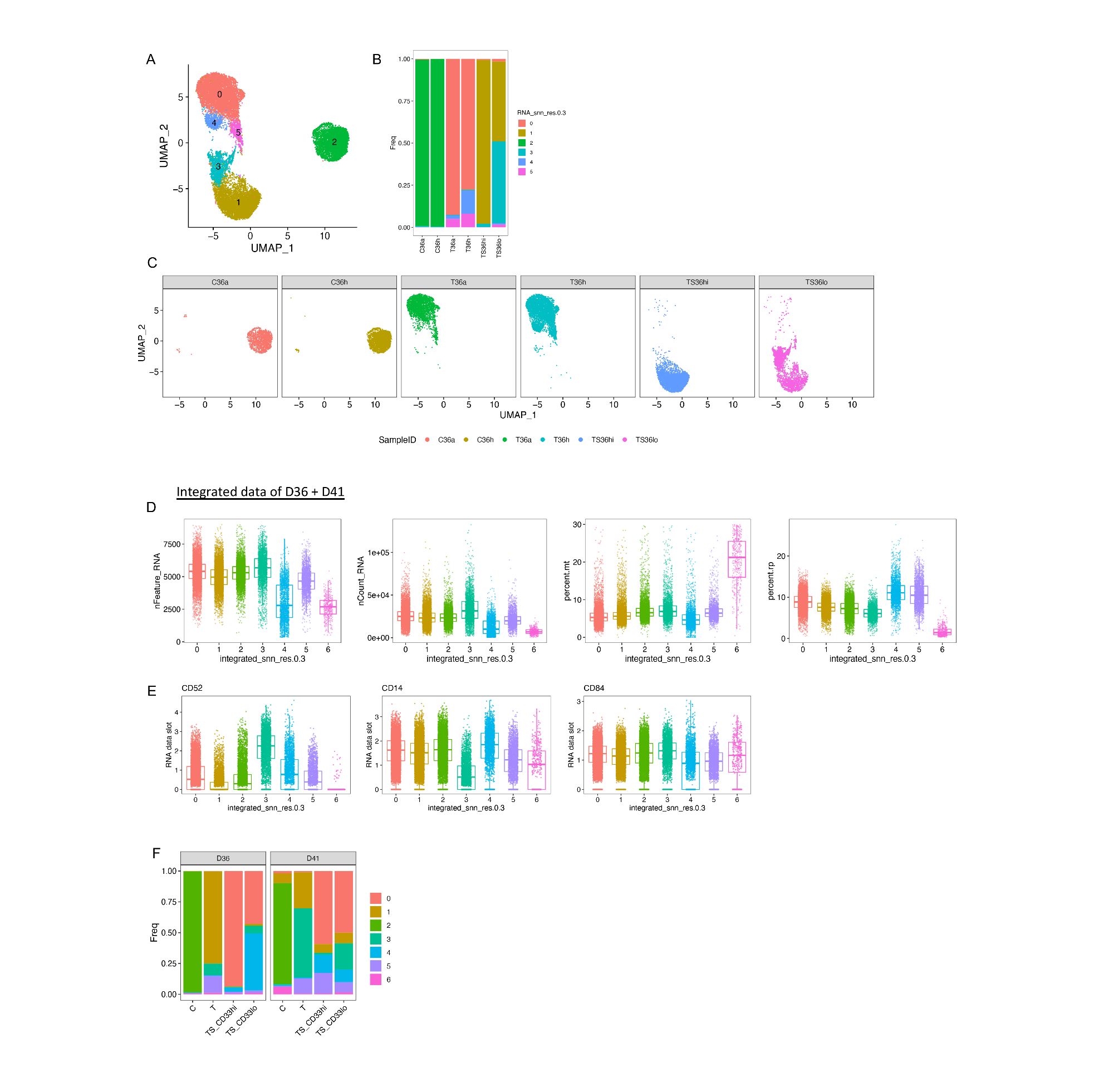


**Fig. S5. Supplemental data for Fig. 2.** (A-C) scRNA-seq results for donor #36. We performed scRNA-seq on sorted Ap+ (appended with “a”, apoptotic) and Ap- (appended with “h”, healthy) cells from the T and C groups, and CD33hi and CD33lo cells from cytokine treated cells after 48h of serum starvation (TS group) from donor #36. (A) UMAP plot showed all the cells passed quality control color coded by identified GEX clusters. (B) The composition of GEX clusters across the six samples. (C) UMAP plot color coded and wrapped by sample ID.

Overall, the transcriptomic patterns in Ap+ and Ap- cells from the same group were quite similar, while we observed a dominant cell subset in CD33lo, that was almost absent in CD33hi. To confirm the differences between the two CD33 populations, we performed another batch of scRNA-seq on the flow isolated monocytes from a different donor, #41. The integrated scRNA-seq data across the two donors were shown in Fig.2. The dominant cell subset in CD33lo in Donor #36 (show in this supplemental Fig. B) was composite of C3 and C4 characterized in Fig. 2G), and only the enrichment of C3 in CD33lo was consistent across donors #36 and #41 by scRNA-seq, which was further validated by flow cytometry across multiple donors.

(D-E) Integrated scRNA-seq supplemental results for donors #36 and #41. The clusters were the same as shown in Fig. 2G. (D) Parameters of quality control across GEX clusters. nFeature_RNA: The number of detected genes. nCount_RNA: The number of detected UMIs (Unique Molecular Identifier). percent.mt: The percentage of UMIs that map to the mitochondrial genome. percent.rp: The percentage of UMIs that map to ribosomal genes. (E) Gene expression of representative markers across GEX clusters. (F) Cell composition of GEX clusters across two donors.


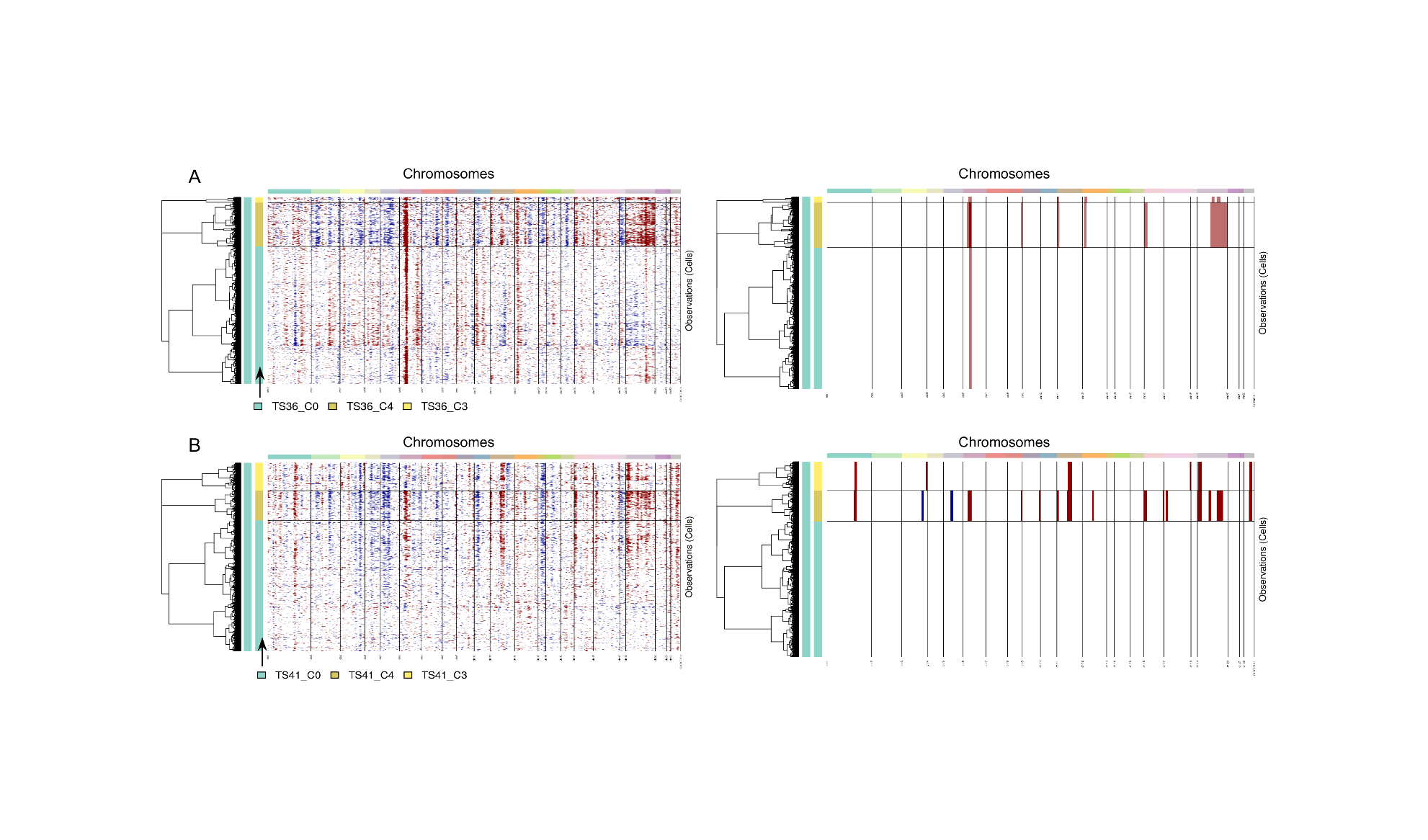


**Fig. S6. Inferred SCNV profile of resistant MDSCs in TS group. Supplemental for Fig. 3**. (A-B) SCNVs were inferred within each cluster of C3, C0 and C4 from TS group. Left panel: The expression values for the genes ordered by genomic locations across the cells from TS group (red: gain vs. blue: loss). Right panel: Predicted SCNVs in C3, C0 and C4. (A) Donor #36. (B) Donor #41.

**
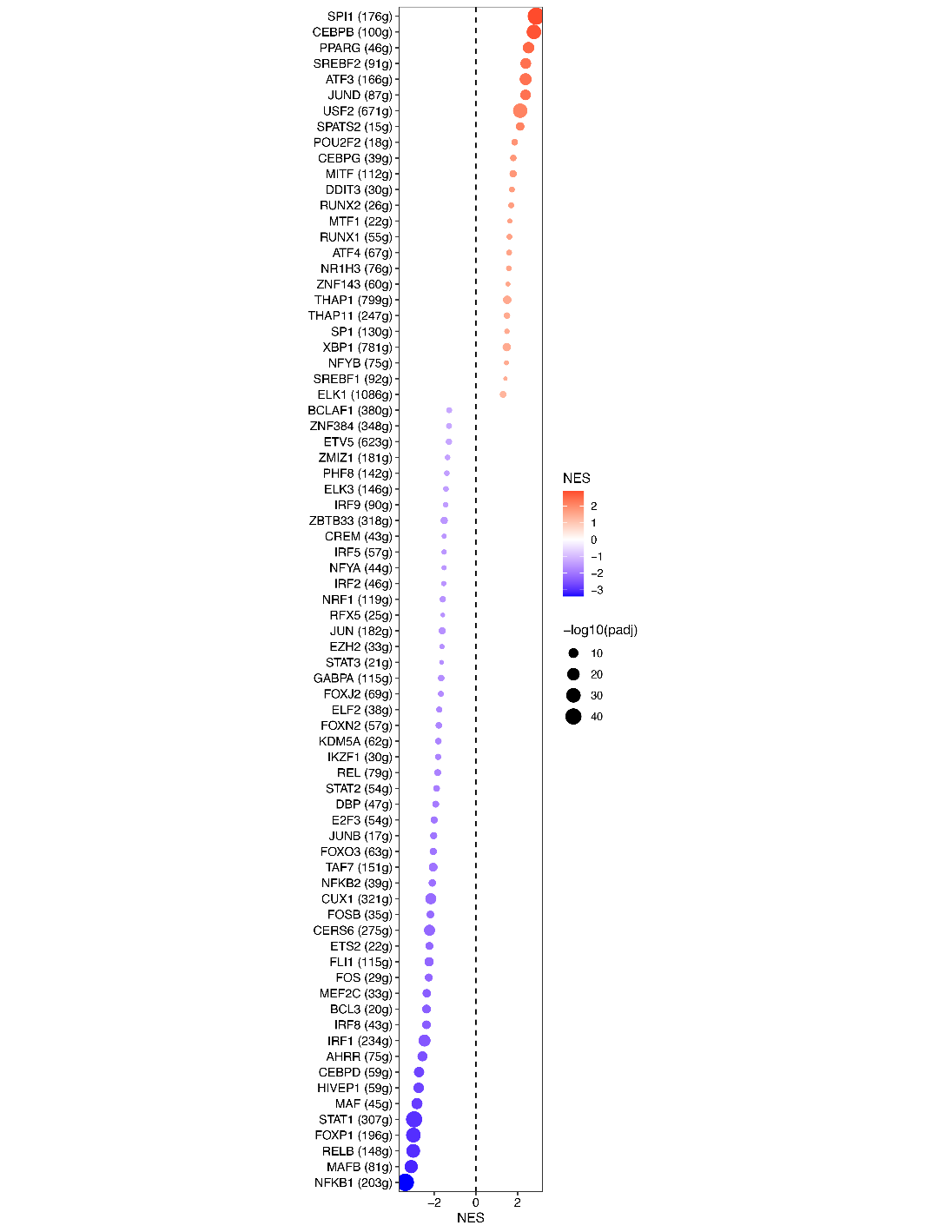
**

**Fig. S7. GSEA results using the genes included in hdWGCNA analysis ranked by kME value for the yellow module. The significant enriched regulons are shown.** Supplemental data for Fig. 4.


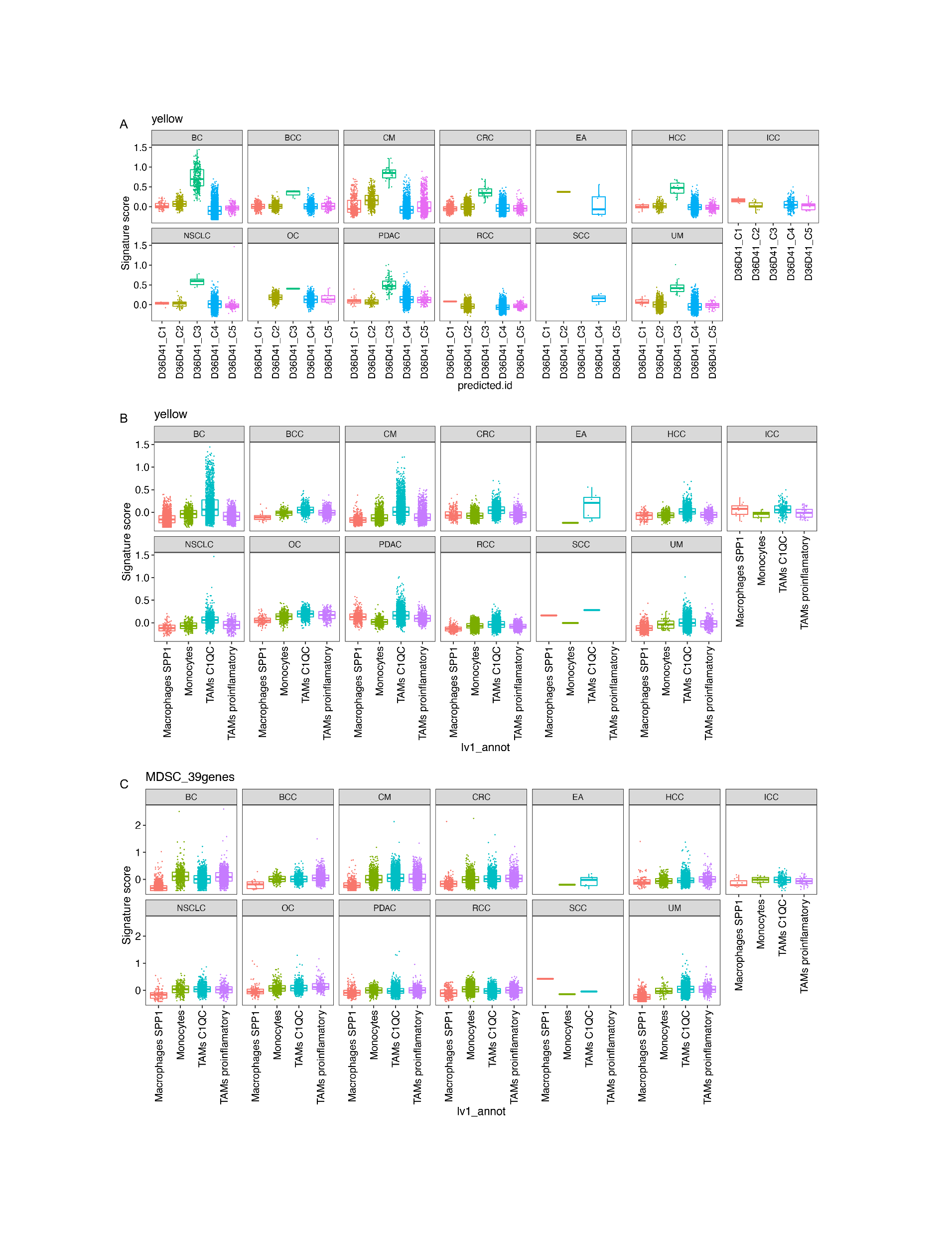


**Fig. S8. Signature scores of different gene sets in the cells belonging to the monocyte-macrophage lineage.** Supplemental data for Fig. 6. (A) Signature scores of the yellow module grouped by the predicted labels. (B) Signature scores of the yellow module grouped by the original cell annotation. (C) Signature score of the public MDSC signature grouped by the original cell annotation.


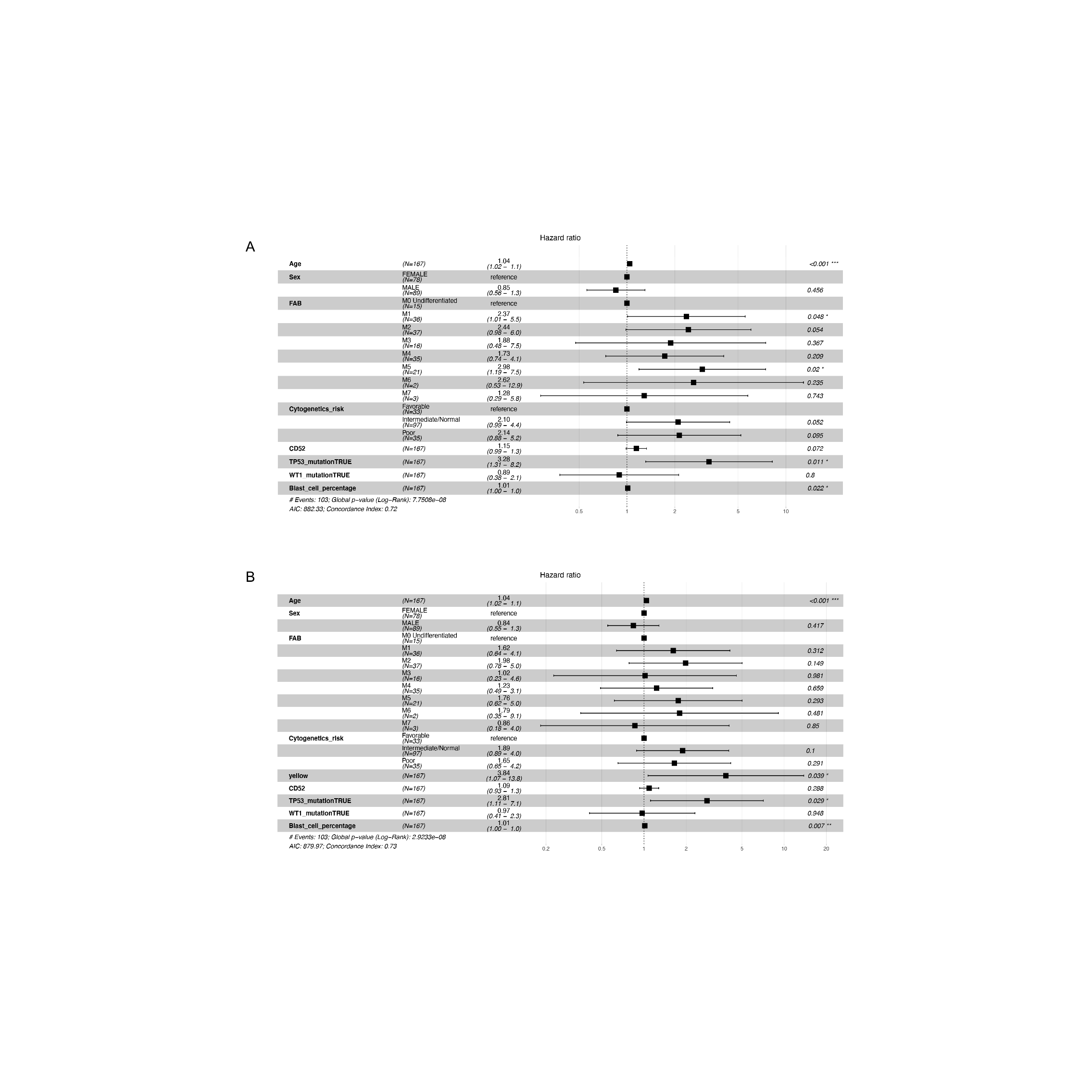


**Fig. S9. Survival analysis using Cox regression model including CD52 gene expression.** **Supplemental for Fig. 7**. (A) Model including CD52 gene expression without the yellow module signature score. (B) Model including both CD52 gene expression and the yellow module signature score.
