# Supplementary material for: Single-cell transcriptomic analysis identified resistant MDSCs and a stress-tolerant gene co-expression network as common MDSC features across multiple disease settings
Source: Front Immunol. 2025 Apr 8;16:1565211. doi: 10.3389/fimmu.2025.1565211 (PMC12011849; doi:10.3389/fimmu.2025.1565211)
Supplement: Supplementary file 5 [file DataSheet2.docx]

**LIST OR REAGENTS**

**Reagents**

10x Genomics single-cell sequencing kits

Chromium Next GEM Single Cell Multiome ATAC + Gene Expression Reagent Bundle (PN-1000283)

Chromium Next GEM Chip J Single Cell Kit (PN-1000234)

Single Index Kit N Set A (PN-1000212)

Dual Index Kit TT Set A (PN-1000215)

Chromium Next GEM Single Cell 3ʹ Kit v3.1 (PN-1000268)

Chromium Next GEM Chip G Single Cell Kit (PN-1000120)

**Antibodies**

anti-human CD33-APC-Cy7: BioLegend, Cat# 303441

anti-human CD11b-Alexa Fluor 647: BioLegend, Cat# 301319

anti-human HLA-DR-PE: BioLegend, Cat# 361605

anti-human CD66b-Pacific Blue: BioLegend, Cat# 305111

anti-human CD52-PE: BioLegend, Cat# 318904

anti-human CD14-PE-Cy7: BioLegend, Cat# 325617

anti-human CD63-PerCP-Cy5.5: BioLegend, Cat# 353020

anti-human CD16-Pacific Blue: BioLegend, Cat# 302024

anti-human CEBP-Beta Alex Fluro 488: Abcam, Cat# ab237414

anti-human CD3-SuperBright 600: Thermo Fisher Scientific, Cat# 63-0037-41

**Purchased frozen cells**

Human Peripheral Blood Mononuclear Cells, Frozen: STEMCELL, Cat# 70025

Human Peripheral Blood Pan-T Cells, Frozen: STEMCELL, Cat# 70024

**DNA-related**

Qiamp DNA Micro kit: QIAGEN, Cat# 56304

Infinium CytoSNP-850K v1.2 BeadChip array: Illumina, Cat# 20025644

**Others**

Human GM-CSF: Sigma-Aldrich, Cat# G5035

Human IL-6: Sigma-Aldrich, Cat# I1395

Accutase: Sigma-Aldrich, Cat# A6964

Human TruStain FcX (Fc receptor blocking solution): BioLegend, Cat# 422302

Live/Dead Aqua Dead Cell Stain dye: Thermo Fisher Scientific, Cat# L34966

Foxp3/Transcription Factor Staining Buffer Set: Thermo Fisher Scientific, Cat# 00-5523-00

UltraComp eBead Plus Compensation Beads: Thermo Fisher Scientific, Cat# 01-3333-42

DCFDA/H2DCFDA-Cellular ROS Assay Kit: ABCAM, ab113851

FITC Annexin V Apoptosis Detection Kit: BioLegend, Cat# 640914

CellTrace Far Red dye: Thermo Fisher Scientific, Cat# C34564

EasySep Human CD33 Positive Selection Kit II: STEMCELL, Cat# 17876

Dynabead Human T-Activator CD3/CD28 for T Cell Expansion and Activation: Thermo Fisher Scientific, Cat# 11161D

Diff-Quik Stain Set: SIEMENS, Cat #B4132-1A

pHrodo Green E. coli BioParticle Conjugate: Thermo Fisher Scientific, Cat# P35366

Complete media: RPMI164+ 10%FBS + penicillin (100U/mL) + streptomycin (100U/mL)

Serum-free media: RPMI164+ penicillin (100U/mL) + streptomycin (100U/mL)

**SUPPLEMENTAL ANALYSIS**

**Infinium CytoSNP-850K v1.2 BeadChip array**

Genomic DNA was extracted from cell pellets using Qiamp DNA Micro kit and processed with CytoSNP array. Allele calling was performed with GenomeStudio 2.0 Genotyping module using default parameters and human reference GRCh38. Data points with GenCall score < 0.15 were deemed unreliable and noted as "No call". Call rates for 16 samples were all > 0.997. For a specific donor, the cytokine-treated sample was designated as subject, and the control sample as reference. We performed paired sample analysis using GenomeStudio and obtained the Paired Sample Table. SNP-based Log2 (Rsub/Rref) values representing the total copy number estimates for treated CD33+ cells relate to the control cells.

**Inferring SCNV profiles using infercnv**

The R package, infercnv (v1.4.0) (1), inputs scRNA-seq data and infers SCNVs in the cells of interest by comparing the gene expression intensities across genomic positions to the reference cells (control CD33+ cells). The genes with the average read counts across cells < 0.1 were excluded. Other parameters were used as default. SCNVs were predicted via six-state hidden Markov model. We inferred SCNVs at the population level to get the inferred SCNVs in each GEX cluster in average. To define SCNV clusters and deconvolute the SCNV-related gene expression, we also ran infercnv under “subcluster” mode regardless of GEX clusters. Because subcluster analysis is computationally intensive, we randomly sampled 1000 cells from each sample for this analysis.

**Cyto-SNP array validated the inferred SCNV profile at DNA level**

To provide data at the DNA-level, we cultured and harvested the samples from eight additional donors. An aliquot of the cells underwent flow cytometry to assess cell surface markers and ROS levels. For ROS detection, cells were stained with 40uM DCFDA at 37° for 30min. The remaining cells were sorted to isolate live CD33+ cells for DNA extraction and CytoSNP microarray for cytogenetics studies (Fig. S3A). For quality control, we first performed PCA based on the SNP-based log2R values across all 16 samples. PC1 captured the differences between males and females, and as expected paired samples from the same donor were closest to each other (Fig. S3B).

To derive the SCNV profiles, we performed PCA using the SNP-based Log2(Rsub:Rref) values that represent the log2 of the ratio of R values from cells from the subject (T group) and reference (C group). To quantify ROS production, we extracted the compensated ROS flow data for live CD33+ cells (Fig. S4A). A Kolmogorov–Smirnov (KS) test was performed between treated vs. control cells from each donor, and the D value was selected to quantify the ROS shift. The D values were significantly correlated with PC1 (Fig. S3C-D) rather than the other PCs (Fig. S4B); therefore, we focused our analysis on PC1. We performed Spearman’s correlation between Log2(Rsub:Rref) values and the PC1 coordinates for each SNP. The SNPs in the amplified regions of SCNV_hi were annotated as SCNV_hi_gain which were correlated with PC1+. The SNPs in the deleted regions were annotated as SCNV_hi_loss and were correlated with PC1- (Fig. S3E). Thus, PC1 recapitulated the inferred SCNV profile with PC1+ associated with SCNV_hi.

**SUPPLEMENTAL METHODS**

**Flow cytometry and sorting**

Cells were incubated with Fc block for 10 min, then stained with antibodies and Live/Dead dye in PBS+2%FBS (FACS buffer) on ice for 30min. Foxp3/Transcription Factor Staining Buffer Set was purchased for intracellular staining, which was performed at room temperature (RT). Cells were permeabilized using 1mL of 1x Fixation/Permeabilization working solution and incubated for 20 min. After washed with 1x Permeabilization buffer twice, cells were stained with intracellular antibodies for 1 hour at RT, and then washed with 1x Permeabilization buffer twice. Cells were finally resuspended in FACS buffer. Single-stained compensation beads and unstained cells were used to set up voltages and compensation parameters. Flow cytometry was performed using BD LSRII. Flow sorting was performed using Aria 5 lasers flow sorter. Data were analyzed using FlowJo. For apoptosis detection, cells were incubated with Annexin V (5uL/million cells) at RT for 15min. Annexin V- gate was defined by Fluorescence Minus One (FMO) control.

**Morphology**

Flow sorted MDSC subsets (CD52^hi^CD14^lo^ vs CD52^lo^CD14^hi^) from CD33^lo^CD11b+ cells after serum starvation) were subjected to cytospin preparations (300 rpm, 3 min) to assess the morphology using Diff-Qui Stain Set. Slides were fixed in fixative solution for 30 sec, stained in solution I for 1 min, then in solution II for 20 sec and finally washed with deionized water. Images were acquired on the Olympus Provis microscope (magnification: 40x).

**Single-cell sequencing raw data processing**

For single-cell multiome or 3’ gene expression kit, the BCL files generated by Illumina sequencing were demultiplexed by *cellranger-arc mkfastq* or *cellranger mkfastq* respectively. We generated feature-barcode count matrix using *cellranger-arc count* or *cellranger count* (mapped to GRCh38 human reference genome) for each sample separately. The outputs of the samples from the same donor were aggregated using *cellranger-arc aggr* or *cellranger aggr*.

**Single-cell Feature-barcode count matrix processing**

*1. Quality control and filtering*

The feature-barcode count matrix was processed using Signac (v1.4.0) (2) and Seurat (v4.0.5) (3). The cells with a total number of RNA features ≤ 200 or extremely high RNA features (cutoff depends on the data distribution) were excluded to remove the cells with low sequencing depth or potential multiplets. The cells with a percentage of mitochondrial genes ≥ 30% were excluded to remove the low-quality cells in the GEX data. The cells with poor nucleosome banding patterns (nucleosome signal ≥ 4) or low TSS enrichment scores (TSS ≤ 2) were excluded to remove the low-quality cells in the ATAC data. The immune cells other than myeloid lineages were also excluded, keeping only myelomonocytic cells for further analysis.

*2. Normalization*

RNA data was normalized using the “LogNormalize” method and scaled by applying linear transformation. ATAC data was normalized by term frequency-inverse document frequency (TF-IDF) normalization followed by singular value decomposition (SVD), known as latent semantic indexing (LSI). We mainly focused on GEX and inferred SCNV profiles, so the single cell data were integrated across different donors only using GEX data to correct for subject heterogeneity.

*3. GEX data integration*

Before data integration, for each donor, RNA data were normalized, and the top 2000 variable features were identified by the “vst” method. The genes that were repeatedly variable across donors were selected, and we performed data integration. We then performed a single analysis (data scaling and PCA) on the integrated RNA data. We performed UMAP analysis and identified cell clusters (called GEX clusters) using the first 30 PCs from the integrated GEX data.

*4. Identification of differential genes, peaks and motifs*

To identify DE genes, we used unintegrated and normalized RNA data with donor as a potential variable. To identify DA peaks, we used unintegrated and normalized ATAC data with the total number of fragments and donors as latent variables. We computed motif activity score for each cell by running chromVAR (4), and differential motifs were identified between groups using computed activity score matrix with donor as a potential variable. Transcription factor binding profiles were obtained from JASPAR2020 R package.

*5. Other secondary analysis*

*PC1 interpretation*:

For each gene, Pearson’s correlation test was performed between the normalized gene expression values and the PC1 coordinates across all single cells. The raw p value was corrected by the Benjamini-Hochberg method to correct for multiple testing. The significant correlated genes (FDR < 0.001 and |r| > 0.1) were ranked by correlation coefficient and used as the input for GSEA.

*Regulon detection*:

Computed using SCENIC (v1.2.4) using default parameters. The computed regulons were further used as the gene sets for enrichment analysis.

*Signature score calculation*:

For the genes of a specific signature, the average of the scaled gene expression values was calculated within each single cell as the signature score.

*Multivariate linear regression*:

To deconvolute the transcriptomic profile associated with a specific variable, we performed gene-based multivariate linear regression. The analysis was performed across the 6000 cells from the cytokine-treated group used in the infercnv subcluster analysis. For each gene, we included four independent variables, including: (1) GEX_PC1 (continuous variable: PC1 coordinates, representing cytokine-induced transcriptomic changes as shown in Fig. 1B), (2) SCNV clusters (categorical variable: SCNV_hi or SCNV_lo as characterized in Fig. S2, Table S1), (3) subject ID (categorical variable: six donor IDs to adjust for subject heterogeneity) and (4) cell cycle phases (categorical variable: G1, G2M and S, computed by Seurat). For a specific variable, the significant genes (FDR < 0.05) were ranked by the corresponding t values as inputs for GSEA for biological interpretation.

Genes significantly associated with SCNV clusters rather than GEX_PC1 were interpreted as SCNV-dependent transcriptional changes. Conversely, genes significantly associated only with GEX_PC1 were interpreted as SCNV-independent transcriptional changes. Genes significantly associated with both SCNV clusters and GEX_PC1 were designated as regulated by both pathways.

*Label transfer:*

We used the scRNA-seq data characterized in Fig. 1 as reference. The cell annotations shown in Fig. 1E were transferred to the scRNA-seq dataset characterized in Fig. 2 using Seurat package. We first identified a set of anchors between the reference and query datasets using FindTransferAnchors() function and the anchors were used to transfer the cell annotations to query object using TransferData() function.

**Statistics**

To identify the differential features (i.e., DE genes) from single-cell data, we used FindMarkers() functions in Seurat package. The raw p value was corrected by Bonferroni correction using all features in the dataset (the default method in Seurat). To quantify the overlap between two gene sets, we used gene set overrepresentation test and computed one-sided hypergeometric p value. The raw p value was corrected by the Benjamini-Hochberg method to correct for multiple testing. We performed GSEA using the fgsea R package (v1.10.1) via fgseaMultilevel() function. The raw p value was corrected by the Benjamini-Hochberg method. For other statistical tests involving multiple testing (i.e., PC1 correlation, multilinear regression), the raw p value was all corrected by the Benjamini-Hochberg method. Adjusted p value < 0.05 was considered significant.

**Characteristics of the donors subjected to omics analysis.**

| **ID** | **Age** | **Sex** | **Source** | **Assay** |
| --- | --- | --- | --- | --- |
| HC3514 | 49 | M | Pitt IRB protocol | single-cell multiome (RNA + ATAC) seq |
| HC3523 | 28 | M | Pitt IRB protocol | single-cell multiome (RNA + ATAC) seq |
| HC3524 | 31 | M | Pitt IRB protocol | single-cell multiome (RNA + ATAC) seq |
| HC3529 | 66 | M | Pitt IRB protocol | single-cell multiome (RNA + ATAC) seq |
| HC3531 | 59 | M | Pitt IRB protocol | single-cell multiome (RNA + ATAC) seq |
| HC3533 | 22 | M | Pitt IRB protocol | single-cell multiome (RNA + ATAC) seq |
| D1 | 50 | F | Purchased from STEMCELL | cytoSNP array |
| D3 | 44 | M | Purchased from STEMCELL | cytoSNP array |
| D4 | 37 | M | Purchased from STEMCELL | cytoSNP array |
| D5 | 49 | F | Purchased from STEMCELL | cytoSNP array |
| D7 | 34 | F | Purchased from STEMCELL | cytoSNP array |
| D8 | 33 | M | Purchased from STEMCELL | cytoSNP array |
| D9 | 35 | M | Purchased from STEMCELL | cytoSNP array |
| D11 | 50 | M | Purchased from STEMCELL | cytoSNP array |
| D36 | 55 | F | Purchased from STEMCELL | single-cell RNA seq |
| D41 | 34 | F | Purchased from STEMCELL | single-cell RNA seq |

**References**

1. inferCNV of the Trinity CTAT Project. <https://github.com/broadinstitute/inferCNV>.

2. Stuart T, Srivastava A, Lareau C, Satija R. Multimodal single-cell chromatin analysis with Signac. 2020:2020.11.09.373613.

3. Stuart T, Butler A, Hoffman P, Hafemeister C, Papalexi E, Mauck WM, 3rd, et al. Comprehensive Integration of Single-Cell Data. Cell. 2019;177(7):1888-902 e21.

4. Schep AN, Wu B, Buenrostro JD, Greenleaf WJ. chromVAR: inferring transcription-factor-associated accessibility from single-cell epigenomic data. Nat Methods. 2017;14(10):975-8.
